# Oligodendroglial somatic *SNCA* copy number gains are associated with inclusions and disease onset in multiple system atrophy

**DOI:** 10.1007/s00401-026-03077-4

**Published:** 2026-09-04

**Authors:** Caoimhe Morley, Ester Kalef-Ezra, Diego Perez-Rodriguez, Zane Jaunmuktane, Christos Proukakis

**Affiliations:** 1 Department of Clinical and Movement Neurosciences, UCL Queen Square Institute of Neurology, London, UK; 2 Queen Square Brain Bank for Neurological Disorders, UCL Queen Square Institute of Neurology, London, UK

**Keywords:** Multiple system atrophy, Synucleinopathy, Somatic mutation, CNV, Mosaicism, Fluorescent in situ hybridisation

## Abstract

Multiple system atrophy (MSA) is a rapidly progressive synucleinopathy of unknown aetiology with neuronal and oligodendroglial α-synuclein inclusions. We previously reported somatic copy number variants (CNVs), specifically gains of *SNCA* (encoding α-synuclein) in MSA brains. Here, we expand on this work by combining fluorescent in situ hybridisation for *SNCA* on nuclei with α-synuclein and SOX10 immunofluorescence, to assess oligodendrocyte-specific *SNCA* gains and losses, and their relationship with inclusions across differentially affected regions in two MSA subtypes: striatonigral degeneration (SND) and olivopontocerebellar atrophy (OPCA). Analysis of 13 SND, 12 OPCA and 15 control brains demonstrated significantly higher somatic *SNCA* CNVs, both gains and losses, in MSA oligodendrocytes compared with controls (gains: 6.3% vs 2.0%; losses: 12.5% vs 7.4%,* p* < 0.0001 for both). Oligodendrocyte *SNCA* gains were high in the preferentially affected regions (putamen in SND, cerebellum in OPCA), where they were associated with a twofold increased presence of α-synuclein inclusions in the same cell (*p* < 0.0001). Higher gain burden correlated with earlier disease onset (rho = − 0.45,* p* = 0.03). Oligodendrocyte *SNCA* losses, conversely, showed less regional predilection, limited association with inclusions and no correlation with onset age. As double-strand DNA breaks have been reported in Lewy body diseases, we used immunofluorescence for γH2AX in a subset of experiments. The proportion of γH2AX-positive cells was significantly higher in MSA than controls overall (4.9% vs 2.5%, *p* = 0.01), in oligodendrocytes (9.7% vs 2.5%, *p* = 0.024) and in other cells (3.2% vs 1.1%; *p* = 0.048). The proportion of γH2AX-positive cells was also higher in preferentially affected regions (6.3% vs 3.5%, *p* = 0.004) and in inclusion-bearing cells (22.2% vs 14.9%,* p* = 0.02). These findings define the oligodendrocyte-specific patterns of somatic *SNCA* CNVs in MSA, support a role for gains in MSA pathogenesis and demonstrate the presence of *SNCA* losses and DNA double-strand breaks which require further investigation.

**Supplementary Information:**

The online version contains supplementary material available at https://doi.org/10.1007/s00401-026-03077-4.

## Introduction

Multiple system atrophy (MSA) is a rare synucleinopathy characterised by varying combinations of autonomic failure, parkinsonism and cerebellar ataxia, and a rapidly progressive disease course [9]. Although neuronal α-synuclein aggregation is a shared hallmark across all synucleinopathies, MSA is uniquely defined by the additional presence of glial cytoplasmic inclusions (GCIs) within oligodendrocytes [13, 66]. Neuronal inclusions, including neuronal cytoplasmic (NCIs) and neuronal nuclear inclusions (NNIs), are distinct from the Lewy bodies (LBs) and Lewy neurites that characterise Parkinson’s disease (PD) and Dementia with Lewy bodies (DLB), which are comparatively rare in MSA [25, 33, 62]. MSA diverges into distinct pathological subtypes, including striatonigral degeneration (SND), olivopontocerebellar atrophy (OPCA) and a mixed subtype. SND predominantly affects the putamen and substantia nigra, whereas OPCA involves the cerebellum, pons and inferior olives, with mixed cases showing overlapping features [16, 29]. Despite these well-defined pathological patterns, the mechanisms that render oligodendrocytes and specific brain regions selectively vulnerable remain unclear and represent a critical gap to bridge in our understanding of MSA pathogenesis and phenotypic heterogeneity.

In contrast to PD and DLB, where genetic studies have identified multiple Mendelian and risk loci implicating diverse biological pathways, the aetiology of MSA is unknown [32]. MSA shows no clear familial aggregation and very low heritability (< 7%) [10], and to date, no major reproducible inherited genetic risk factors have been established [7, 64]. Nevertheless, chronic elevation of α-synuclein is sufficient to drive its aggregation, as demonstrated in PD cases with germline *SNCA* multiplications [19]. These cases highlight the strong dosage sensitivity of α-synuclein: *SNCA* duplications typically lead to later-onset parkinsonism with reduced penetrance, whereas triplications cause early-onset, rapidly progressive disease [19]. Some individuals with *SNCA* multiplications also exhibit mixed neuronal and glial pathology, suggesting that excess α-synuclein expression can also be detrimental to oligodendrocytes [73]. Experimental models further support this, as oligodendrocyte-specific overexpression of human α-synuclein in mice generates GCI-like pathology, demyelination and neuroinflammation, with higher expression producing earlier and more severe neurodegeneration [59, 70]. Together, these observations suggest that cell- or region-restricted increases in *SNCA* dosage could contribute to the pathogenesis of MSA. Therapeutic approaches that lower α-synuclein levels, including antibody-based and antisense strategies, are already in development [3].

Somatic mutations, arising post-zygotically and resulting in genetic mosaicism within the brain, have emerged as potential contributors to neurodegenerative disease [35, 41, 50, 68]. These alterations encompass a wide range, from single nucleotide variants to large-scale copy number variants (CNVs, gains or losses) and other structural variants. Somatic mutations may result from mitotic errors or diverse types of DNA damage, with double-stranded DNA breaks (DSBs) being the most severe form. DSB repair in post-mitotic cells is primarily by non-homologous end joining (NHEJ) [36] which can lead to deletions [53]. DSBs have been reported in Lewy body diseases [31, 63], but not investigated in MSA to our knowledge. We previously demonstrated through fluorescence in situ hybridisation (FISH) studies of somatic CNVs, specifically gains, of *SNCA* (encoding α-synuclein) in substantia nigra dopaminergic neurons in PD, with the highest levels reported in two MSA cases [43]. Subsequent work detected higher levels of *SNCA* gains in neurons of the cingulate cortex in both PD and MSA compared to controls, and in non-neurons in MSA only [47]. More recently, we demonstrated increased levels of somatic *SNCA* gains in MSA-affected regions, the putamen in SND and cerebellum in OPCA, with an increased relative risk for a-synuclein inclusions at the single-cell level [14].

Importantly, these studies did not establish whether *SNCA* gains occur in oligodendrocytes, although their level was higher in oligodendrocytes in the substantia nigra of three SND cases analysed. Moreover, none included region-matched controls from the putamen, cerebellum and substantia nigra, precluding assessment of whether somatic *SNCA* gains in these regions are truly enriched in MSA. Finally, the potential role of somatic *SNCA* losses has been unexplored and may have distinct functional consequences. Loss of α-synuclein function is not a known driver of any neurodegenerative disease, and experimental models have produced conflicting results regarding its impact [1, 6, 26, 52]. However, aggregated α-synuclein may sequester the protein and diminish its normal physiological functions [58, 69], raising the possibility that dosage reductions could contribute to cellular vulnerability under certain conditions. Together, these observations highlight the need for a systematic, cell-type-resolved and region-matched assessment of somatic *SNCA* CNVs in MSA.

Here, we aim to address these gaps by applying an optimised protocol combining FISH and immunofluorescence on isolated nuclear suspensions, to eliminate sectioning artefacts and reliably call copy number losses in addition to gains. We find that, in MSA, somatic *SNCA* gains are enriched in SOX10^+^ oligodendrocytes relative to controls, in subtype-specific vulnerable regions, and associate with α-synuclein inclusions at both single-cell and regional levels, consistent with a role in pathogenesis. A higher burden of oligodendrocyte *SNCA* gains correlates with earlier disease onset, supporting a model in which pre-existing somatic *SNCA* mosaicism may predispose individuals to α-synuclein aggregation. We now show, for the first time, somatic *SNCA* losses, which are also higher in MSA, though more broadly distributed, with less clear regional associations with inclusions and no association with age of onset. Our results provide further insights into somatic *SNCA* CNVs in MSA, with oligodendrocyte gains as potential drivers of pathogenesis. Furthermore, we perform immunofluorescence staining of tissue sections for phosphorylated H2AX at serine 139 (γH2AX), an established DSB marker [37], which reveals increased DSBs in MSA compared to controls, notably in oligodendrocytes and in cells with α-synuclein inclusions.

## Materials and methods

### Human fresh-frozen post-mortem brain tissue

Fresh-frozen post-mortem brain samples were obtained from the Queen Square Brain Bank for Neurological Disorders in London, United Kingdom. All subjects or their next of kin had given informed consent for brain donation and use in research. Ethics approval was provided by the NHS Health Research Authority Ethics Committee London-Central (REC reference 23/LO/0044). Tissue chips and 10 μm tissue sections were obtained from three brain regions including cerebellar white matter, putamen at the level of the anterior commissure, and the substantia nigra at the level of the red nucleus or decussation of the superior cerebellar peduncle. Although tissue sampling was focussed on cerebellar white matter, adjacent cerebellar cortex may have been sampled due to limitations in dissection. For clarity, these are hereafter referred to as the *putamen, cerebellum* and *substantia nigra*, respectively. For the flow cytometry experiment, additional tissue from the internal capsule was obtained from one control and one MSA-SND. This study included a total of 42 subjects: 27 with pathologically confirmed MSA (15 SND, 12 OPCA) and 15 controls (14 neurologically healthy, 1 with dystonia but normal neuropathology). A summary of experiments performed across each group and brain region is shown in Supplementary Table 1.

### Nuclei isolation from fresh-frozen post-mortem brain tissue

Nuclear fractions were isolated from fresh-frozen tissue as previously described [23, 47, 48]. All procedures were performed at 4 °C to maintain nuclei integrity. Briefly, 20–50 mg of tissue was added to 500 μL of Nuclear Isolation Medium (NIM: 25 mM KCl, 5 mM MgCl_2_, 10 mM Tris/HCL pH 8.8, 250 mM sucrose, 1X cOmplete™ EDTA-free Protease Inhibitor Cocktail (Roche, 04693132001) and 1 mM dithiothreitol. Tissue was initially homogenised using a plastic pestle (20 strokes) and supplemented with 0.1% Triton-X 100. Complete homogenisation was performed using Dounce tissue grinders with a loose and tight pestle (5–10 strokes each, Sigma-Aldrich D8938). The homogenate was centrifuged at 1000* g* for 8 min at 4 °C, after which the supernatant was removed. The resulting pellet was resuspended in 700 μL of 25% Iodixanol prepared using 417 μL of Optiprep™ Density Gradient Medium (60% w/v Iodixanol, Sigma-Aldrich D1556) in 500 μL of NIM and 83 μL of Optiprep Diluent for Nuclei (ODN: 150 mM KCl, 30 mM MgCl_2_, 60 mM Tris/HCl pH 8.8 and 250 mM sucrose, 1X cOmplete™ EDTA-free Protease Inhibitor Cocktail). Subsequently, the 25% Iodixanol/homogenate mixture was gently layered using a syringe onto 1 mL of 29% Iodixanol (483 μL Optiprep™ Density Gradient Medium and 517 μL ODN) to form a gradient. The tube was ultracentrifuged at 10,300* g* for 20 min at 4 °C. The debris layer and supernatant were discarded, leaving 50 μL of buffer above the pellet. The pellet was resuspended according to the downstream analysis (see subheadings Combined immunofluorescence in situ hybridisation (Immuno-FISH) and flow cytometry).

### Combined immunofluorescence in situ hybridisation (immuno-FISH) on isolated nuclei

We used the previously published FISH protocol on tissue sections [15] with minor modifications for compatibility with isolated nuclei. The same custom-designed red 50 kb *SNCA* probe SureFISH (Agilent G110997R-8) was used, and in a subset of experiments, a green 767 kb Chr 7 chromosome enumeration probe (CEP) (G110899G-8) was included for reference. To account for potential variability between experiments, each batch was prepared containing a mixture of MSA subtypes and controls.

Isolated nuclei were fixed with ice-cold Carnoy’s Fixative (3:1 Methanol: Acetic Acid) and incubated on a rotator disk for 1 h at 4 °C. The nuclei were dropped onto an Epredia™ SuperFrost Ultra Plus Gold Adhesion slides (Epredia, 11,976,299) and allowed to fully evaporate. Nuclei were permeabilised using a solution of 0.005% pepsin (Roche, 10,108,057,001) in 10 mM HCl at 37 °C for 5 min. Following digestion, slides were transferred to PBS supplemented with 1 mM MgCl₂ for 5 min, then washed twice in PBS. Slides were dehydrated through an ethanol series consisting of 70%, 90% and 100% molecular-biology grade ethanol, each for 2 min, and then air-dried for 10 min. Next, slides were treated with 70% UltraPure™ formamide (Invitrogen, 15,518,746) in 2X saline sodium citrate (SSC) buffer at 72 °C for 2 min. A second dehydration step was performed using ice-cold ethanol solutions (70%, 90%, and 100%).

FISH probe reactions were prepared according to the manufacturer’s guidelines. The FISH probe mixture was denatured at 72 °C for 5 min, and 10 µL of the mixture was applied per slide. A 22 × 22 mm coverslip was mounted and sealed using Fixogum rubber cement. Hybridisation was carried out in a humidified chamber at 37 °C for 48–72 h. After hybridisation, slides were immersed in 2× SSC buffer at room temperature (RT) for 10 min to allow coverslip removal. They were then washed in preheated Wash Buffer 1 (Agilent, G9401A) at 72 °C for 2 min, followed by a 1 min wash in Wash Buffer 2 (Agilent, G9402A) at RT. Finally, slides were washed three times in PBS for 10 min each.

For immunostaining, blocking solution was prepared with 10% goat serum in PBS with 0.2% Triton-X 100 and incubated for 1 h at RT. Primary antibody solutions were prepared in 2% goat serum and 0.2% Triton-X 100 according to the concentrations listed (see subheading *Antibodies used*). All primary antibodies were incubated overnight at 4 °C. Following washes, species-specific secondary antibodies were applied and incubated for 1 h at RT. Nuclei were counterstained with 1 µg/mL DAPI (4',6-diamidino-2-phenylindole) solution for 20 min. Slides were cover-slipped with Agilent Mounting Buffer (G9403A).

Slides were imaged using a Leica DM6B epifluorescence microscope coupled to an ORCAII Digital CCD camera (Hamamatsu, Shizuoka, Japan) and controlled by Leica Application Suite X (Leica, Wetzlar, Germany). Z-stacks of 10 images separated by 0.5 μm stacks (5 μm depth total) using appropriate fluorescence filters were obtained with a 40X lens objective. All images were analysed blinded to the donor group and region. FISH signals were visualised using the ImageJ software v1.53t and analysed through manual counting first without the protein markers to obtain unbiased counts and then reviewed with the other colour channels. Exclusion criteria for a cell included (1) autofluorescence, as identified by overlapping signals on more than one channel, obscuring the nucleus, (2) faint, unclear or completely absent FISH signals, and (3) indistinguishable copy number status of overlapping nuclei.

### Immunofluorescence on fresh-frozen tissue sections

Fresh-frozen tissue sections were cryosectioned at 10 μm thickness and mounted onto Superfrost Plus slides. Tissue sections were thawed for 20 min at RT and then fixed with 10% neutral-buffered formalin for 20 min at RT followed by permeabilisation with 0.3% Triton-X in PBS for 60 min. A blocking solution containing 10% goat serum in 0.2% Triton-X 100 in PBS was applied for 1 h at RT. Primary antibodies were prepared in 2% goat serum and 0.2% Triton-X 100 according to the concentrations listed (see subheading *Antibodies used*) and incubated overnight at 4 °C. Secondary antibodies were prepared in 2% goat serum and 0.2% Triton-X 100 and incubated for 1 h at RT. A 1X solution of TrueBlack® Lipofuscin Autofluorescence Quencher (Biotium, 23,007) was prepared in 70% ethanol and was applied to each slide for 1 min. DAPI (4′,6-diamidino-2-phenylindole) was applied at a final concentration of 1 µg/mL to each slide for 20 min. Slides were mounted with ProLong™ Gold Antifade (Thermo Fisher Scientific, P36930).

For immunofluorescence markers, image analysis was performed through manual counting using ImageJ software v1.53t. Individual optical Z-stacks (10 stacks at 0.5 μm intervals, corresponding to a total optical depth of 5 μm) were examined to confirm true cellular co-localisation and exclude artefacts arising from overlapping fluorescence in adjacent focal planes. Cell counts and inclusion co-localisation were performed manually within defined regions of interest using merged and single-channel images. Identical exposure and threshold settings were applied across all samples within each staining batch.

### Flow cytometry

Isolated nuclear pellets were gently resuspended in 500 μL of pre-chilled blocking buffer (PBS supplemented with 10% goat serum and 1X cOmplete™ EDTA-free Protease Inhibitor Cocktail, Roche 04693132001). Nuclei were incubated for 30 min at 4 °C on a rotator to block nonspecific binding. For primary antibody staining, nuclei were incubated overnight at 4 °C with continuous rotation. Negative controls were prepared with a secondary only antibody control by aliquoting 50uL of the nuclear suspension into a separate tube and bringing the volume to 500uL with blocking buffer. Secondary antibodies were added directly to the blocking buffer and incubated for 1 h at 4 °C with continuous rotation. DAPI (0.1 μg/mL) was added for 30 min to enable nuclear identification. Fluorophores with well-separated spectra, Alexa Fluor 488 and 647, were selected to minimise spectral overlap. Following staining, nuclei were pelleted by centrifugation (800 × g, 10 min, 4 °C), washed with PBS twice, and resuspended in sorting buffer (PBS containing 5 mM EDTA). To remove aggregates and debris, suspensions were passed through a 70 μM cell strainer prior to flow cytometric sorting. Flow cytometry analysis was performed using a BD FACSAria™ Fusion cell sorter (UCL Paul O’Gorman Cancer Institute Flow Cytometry Core Facility). The gating strategy included: (1) FSC-A vs SSC-A to identify all events; (2) FSC-A vs FSC-H and (3) SSC-A vs SSC-W to exclude doublets; (4) DAPI-positive gate to select intact nuclei; and (5) fluorophore channels corresponding to the antibodies used (Alexa Fluor 488 and Alexa Fluor 647) to resolve specific subpopulations.

### Representative diagnostic FFPE sections

Sections of 7 μm were cut from a formalin-fixed, paraffin-embedded (FFPE) tissue block collected as part of the routine diagnostic neuropathological examination. They were mounted on glass slides and immunostained for α-synuclein on an automated Ventana staining platform (Roche). Detection was carried out using biotinylated secondary antibodies, a horseradish peroxidase–streptavidin complex, and diaminobenzidine (DAB) as the chromogen, followed by haematoxylin counterstaining. Slides were digitised on Hamamatsu S360 slide scanner at ×40 resolution and images prepared from the digital scan.

### Antibodies used

FFPE staining was performed using a mouse monoclonal antibody (MA1-90,342, Thermo Scientific; 1:1,500). The antibody dilutions described below were used for immunofluorescence on frozen tissue sections, immunofluorescence on nuclear suspensions and flow cytometry. To label oligodendrocytes, a rabbit monoclonal anti-SOX10 antibody (1:50; 0.5 µg/mL; clone SP267, Abcam, ab227680) was used. Alpha-synuclein inclusions were detected using either a mouse monoclonal antibody (1:100; 2 µg/mL; clone 211, Santa Cruz, sc-12767) or a rabbit monoclonal antibody (1:1000; 1 µg/mL; clone MJFR1, Abcam, ab13850), depending on the compatibility with co-stained markers. A mouse monoclonal anti-phospho-Histone H2AX (Ser139) antibody (1:200; 5 µg/mL; clone JBW301) was used to detect DNA DSB repair. Secondary antibodies (anti-mouse/anti-rabbit) were used conjugated to 488 and 647 fluorophores (Thermo Fisher Scientific) at 2 μg/mL (1:500).

### Statistics

All statistical analyses were performed in RStudio v2024.12.1 unless otherwise stated. Variables were assessed for normality using the Shapiro–Wilk test, and equality of variance was assessed with Levene’s test for homogeneity of variance. Pairwise comparisons of normally distributed data were performed using the Student *t *test (with Welch correction for unequal variance) or the non-parametric Mann–Whitney U test accordingly. For comparisons involving more than two groups, a one-way ANOVA for normally distributed data was used with Tukey’s HSD post hoc correction, and the non-parametric Kruskal–Wallis was used with Dunn’s post hoc correction. Where applicable, Bonferroni correction was applied to adjust for multiple comparisons. Contingency tables were analysed using the Chi-square test or Fisher’s Exact test. For correlation assessments, Spearman’s rank correlation was performed. Where applicable, Bonferroni correction was applied to adjust for multiple comparisons. Associations between *SNCA* copy number status and α-synuclein inclusions were assessed using a generalised linear mixed-effects model with a binomial error distribution. *SNCA* copy number status was included as a fixed effect and donor as a random effect to account for repeated measurements from the same individual. Results are reported as odds ratios (ORs) and Wald *p *values. For γH2AX analysis, a linear regression was performed using PMI as a covariate. For normally distributed data, results are presented as the mean ± standard deviation (SD); for non-normal data or non-parametric comparisons, the median and interquartile range (IQR) are reported. Box plots display the median and interquartile range, with whiskers extending to 1.5× the interquartile range. Contingency tables were analysed using the Chi-square test or Fisher’s Exact test. For correlation assessments, Spearman’s rank correlation was performed. Unless otherwise stated, *p *values are nominal and two-tailed.

## Results

### Somatic *SNCA* CNVs are more frequent in MSA than controls and gains are enriched in MSA oligodendrocytes

**Fig. 1:**
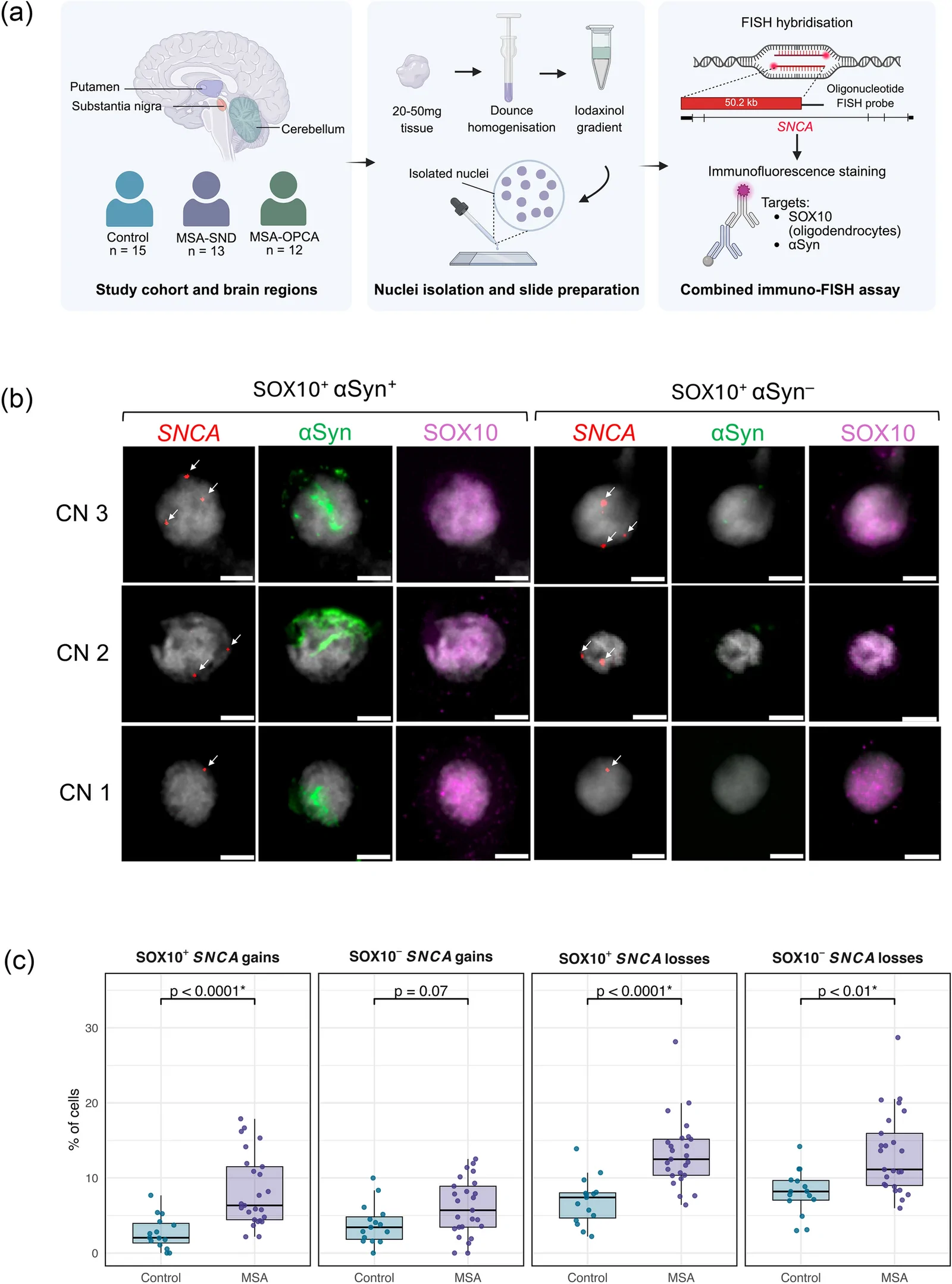
**a** Experimental overview of the immuno-FISH workflow. Fresh-frozen post-mortem tissue from control donors and individuals with SND and OPCA was processed to generate isolated nuclear suspensions. Tissue was dissociated by Dounce homogenisation and nuclei were separated through an Iodixanol gradient before mounting on slides. The combined immuno-FISH assay used an *SNCA* oligonucleotide FISH probe together with immunofluorescent detection of SOX10 to mark oligodendrocyte lineage nuclei and α-synuclein to detect inclusions. Created in BioRender. M, C. (2026) https://BioRender.com/xtqohau**b** Representative immuno-FISH images showing *SNCA* FISH signal together with α-synuclein and SOX10 staining. Examples of nuclei with different *SNCA* copy number (CN) states are shown for SOX10^+^ α-synuclein^+^ and SOX10^+^ α-synuclein⁻ nuclei. White arrows indicate *SNCA* FISH spots. Scale bar = 5 μm. **c** Quantification of somatic *SNCA* CNVs in SOX10^+^ and SOX10⁻ nuclei across MSA (*n* = 25) and control (*n* = 15) groups. Box plots display the proportion of nuclei with *SNCA* gain or loss within each group. Group comparisons were performed with the Mann–Whitney *U* test

To explore whether somatic *SNCA* CNVs are associated with inclusion-bearing cells in MSA, we performed FISH using a custom-designed SureFISH 50 kb *SNCA* probe we previously used [14, 47], combined with immunofluorescence for SOX10, a well-established oligodendrocyte marker [49, 60], which is expressed across oligodendrocyte maturation stages [18], and α-synuclein to visualise inclusions. To characterise somatic losses of *SNCA*, which cannot be reliably done on tissue sections due to sectioning artefacts, all experiments were done on isolated nuclei from fresh-frozen post-mortem brain tissue (workflow depicted in Fig. 1a). FISH on isolated nuclei allowed us to also significantly reduce the pepsin exposure time, a type of protease, which is known to variably digest inclusions in MSA [71], therefore allowing for better epitope preservation. We found that, in MSA, α-synuclein inclusions are well-retained in nuclear preparations due to their perinuclear and/or nuclear localisation (Supplementary Fig. 1a), with no significant difference observed between levels of inclusions on sections versus nuclei as detected by immunofluorescence for the same case and region (*p* = 0.37, n = 8, Supplementary Fig. 1b). We note that in most cases, the proportion of inclusions appeared slightly higher than in sections, which most likely represents sampling bias. To provide further evidence of retention of inclusions in nuclear preparations, we performed flow cytometry combining SOX10 and α-synuclein staining in the cerebellum and internal capsule of one MSA case and one control (Supplementary Fig. 1c,d). This revealed α-synuclein staining nuclei only in MSA and allowed us to estimate an inclusion proportion in SOX10^+^ cells of 15.4% and 3.9% respectively.

The final dataset included at least one brain region from 13 SND cases, 12 OPCA cases and 15 controls. All three brain regions (putamen, cerebellum, substantia nigra) were analysed in ten controls, six SND and seven OPCA cases. There were no statistically significant differences in mean age at death across groups (Table 1) (controls: 71.3 years; SND: 67.9 years; OPCA: 64.2 years, *p* = 0.14) or in median post-mortem interval (PMI) (controls: 46.6 h; SND: 43.8 h; OPCA: 67.5 h, *p* = 0.12). Sex distribution was comparable between groups (*p* = 0.83). Amongst MSA subtypes, there were no significant differences in mean age at disease onset (SND: 61.8 years; OPCA: 57.1 years, *p* = 0.14) or in disease duration (SND: 6.2 years; OPCA: 8.0 years, *p* = 0.10). The overall median total cell count per region from each brain was 88, and the median SOX10⁺ cell count was 46. No significant differences were observed in total cell counts (*p* = 0.4) or SOX10⁺ cell counts (*p* = 0.08) (Supplementary Fig. 2).

**Table 1:** Summary of donor characteristics for the immuno-FISH cohort

Group	n	Age at death (years)		PMI (hrs)		Age at disease onset (years)		Disease duration (years)		Sex (M/F)
		Mean ± SD	Median [IQR]	Mean ± SD	Median [IQR]	Mean ± SD	Median [IQR]	Mean ± SD	Median [IQR]	
Control	15	71.3 ± 11.2	71 [63–75]	64.1 ± 43.4	46.6 [38.6–96.5]	n.a	n.a	n.a	n.a	8 (53.3%) / 7 (46.75)
SND	13	67.9 ± 8.3	67 [63–72]	49.8 ± 22.1	43.8 [36.8–53]	61.8 ± 8.0	63 [57–67]	6.2 ± 1.3 (4–8)	6 [5–7]	6 (46.2%) / 7 (53.8%)
OPCA	12	64.2 ± 6.8	66.5 [57.8 – 69.2]	69.3 ± 21.1	67.5 [58.4–86]	57.1 ± 6.5	57 [52–61.5]	8.0 ± 3.3 (4–14)	7 [6–10]	5 (41.7%) / 7 (58.3%)
p value		ANOVA (*p* = 0.14)		Kruskal–Wallis (*p* = 0.12)		Student t-test (*p* = 0.14)		Student t-test (*p* = 0.10)		Chi-square (*p* = 0.8)

Demographic and sample characteristics of control and MSA subgroups. Data are presented as mean ± SD (standard deviation) and median [interquartile range]. PMI, post-mortem interval. SND, striatonigral degeneration. OPCA, olivopontocerebellar atrophy. n.a. = not applicable

All results divided by disease status, brain region and cell type are shown in Supplementary Table 2. Indicative images of oligodendrocytes with and without CNVs and inclusions are shown in Fig. 1b. We first quantified the percentage of SOX10⁺ cells with *SNCA* gains in both MSA subtypes and controls by taking the average across the regions analysed. This revealed a significant difference in the percentage of SOX10⁺ gains in MSA, where they were enriched over threefold compared to controls (6.3% vs 2.0%, *p* < 0.0001). *SNCA* gains in SOX10⁻ cells were also higher (5.7 vs 3.4%); however, this was non-significant (*p* = 0.07) (Fig. 1c). We next assessed somatic *SNCA* losses, which were even higher than gains, and significantly increased in MSA compared to controls in both SOX10^+^ cells (12.5% vs 7.4%,* p* < 0.0001) and SOX10⁻ cells (11.1 vs 8.2%, *p* < 0.01). These results indicate that somatic *SNCA* gains and losses are both enriched in MSA, preferentially in oligodendrocytes, with less striking enrichment across SOX10⁻ cells.

### Reference probe locus exhibits relative genomic stability in MSA compared to controls and also compared to *SNCA*

**Fig. 2:**
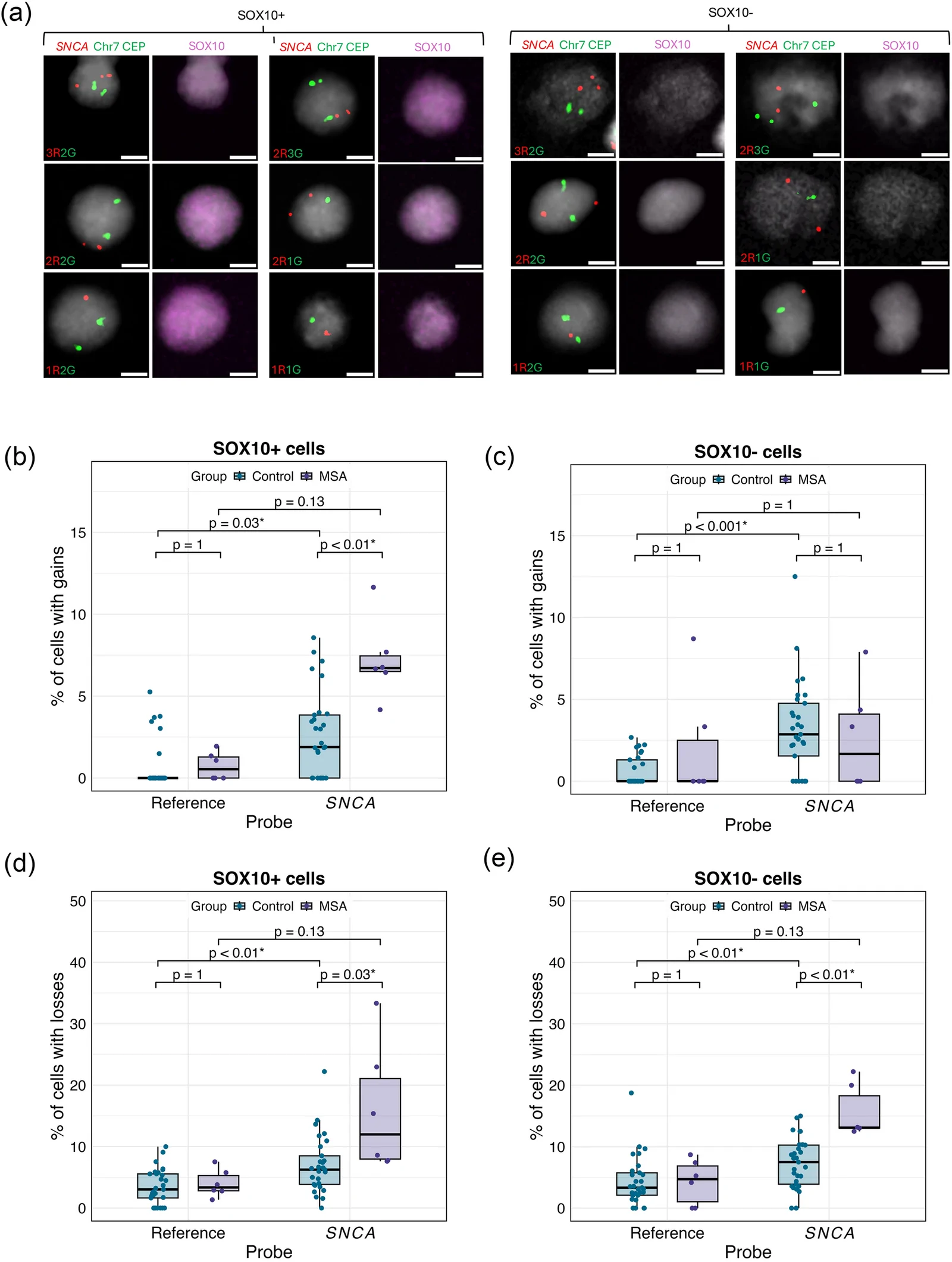
Two-probe FISH analysis across SOX10^+^ oligodendrocytes and SOX10⁻ cells: (**a**) representative immuno-FISH images showing examples of *SNCA* and reference probe with SOX10 staining and different copy number states. Scale bar = 5 μm, (**b**) reference probe and *SNCA* gain quantification in SOX10^+^ cells, (**c**) reference probe and *SNCA* gain quantification in SOX10⁻ cells, (**d**) reference probe and *SNCA* loss quantification in SOX10^+^ cells, (**e**) reference probe and *SNCA* loss quantification in SOX10⁻ cells. Analyses were performed on MSA (6 regional observations from 3 donors) and control (29 regional observations from 14 donors). Statistical significance was determined using Mann–Whitney U test and paired Wilcoxon signed-rank tests, with Bonferroni correction for four comparisons

To assess whether the observed *SNCA* CNVs reflect a locus-specific instability rather than a global genomic phenomenon, we omitted the α-synuclein staining and used a reference probe targeting chromosome 7 for most control experiments), and a subset of MSA (representative images in Fig. 2a). SOX10⁺ reference gains were rare and comparable between MSA and controls (median 0% for controls, 0.5% for MSA, adjusted* p* = 1), (Fig. 2b) as were SOX10⁻ gains (median 0% for both, adjusted* p* = 1) (Fig. 2c). Reference losses were higher but also showed no significant differences between MSA and controls for SOX10⁺ cells (median 3.4% vs. 3.0%, adjusted* p* = 1) (Fig. 2d) or SOX10⁻ cells (median 4.7% vs. 3.3%, adjusted* p* = 1) (Fig. 2e). Within SOX10⁺ cells, gains of both probes were found in only one cell in the MSA case and none in the control group. Amongst SOX10⁻ cells, gains of both probes were identified in a small number of control cells and were not detected in MSA. The median proportions of cells carrying both-probe losses were higher than for gains but were comparable between MSA and controls (SOX10⁺ = 1.2% vs 1.7%, SOX10⁻ = 1.3% vs 1.7%).

Next, to directly compare *SNCA* with the reference locus, we performed paired analyses within the same case and region. In controls, *SNCA* gains were significantly more frequent than reference gains in both SOX10^+^ (1.9% vs 0%, adjusted* p* = 0.03) (Fig. 2b) and SOX10⁻ populations (2.9% vs 0%, adjusted* p* < 0.001) (Fig. 2c). In MSA, the same trend was observed in SOX10^+^ gains, though the difference did not reach significance after correction (6.7% vs 0.5%, adjusted* p* = 0.13) (Fig. 2b). There was no difference between SOX10⁻ *SNCA* and reference gains (1.7 vs 0%, adjusted* p* = 1) (Fig. 2c). A similar pattern was observed for losses. In controls, *SNCA* losses were significantly more frequent than reference losses in both SOX10⁺ (median = 6.3% vs. 3.0%; adjusted* p* < 0.01) (Fig. 2d) and SOX10⁻ populations (median = 7.5% vs. 3.3%; adjusted* p* < 0.01) (Fig. 2e). In MSA, *SNCA* losses were likewise higher relative to reference losses in SOX10⁺ cells (median = 12.0% vs. 3.4%) though this did not reach significance (adjusted* p* = 0.13) (Fig. 2d). Similarly, in SOX10⁻ cells, there was an increase in *SNCA* losses compared to the reference though this was non-significant (median = 13.1% vs. 4.7%; adjusted* p* = 0.13) (Fig. 2e). Overall, these results support the relative genomic stability of the reference probe locus, consistent with our previous findings [14, 43, 47] and indicate that CNVs are preferentially enriched at the *SNCA* locus, including both gains and losses in controls.

### Somatic *SNCA* gains in oligodendrocytes are most enriched in severely affected MSA regions and correlate with α-synuclein inclusions

**Fig. 3:**
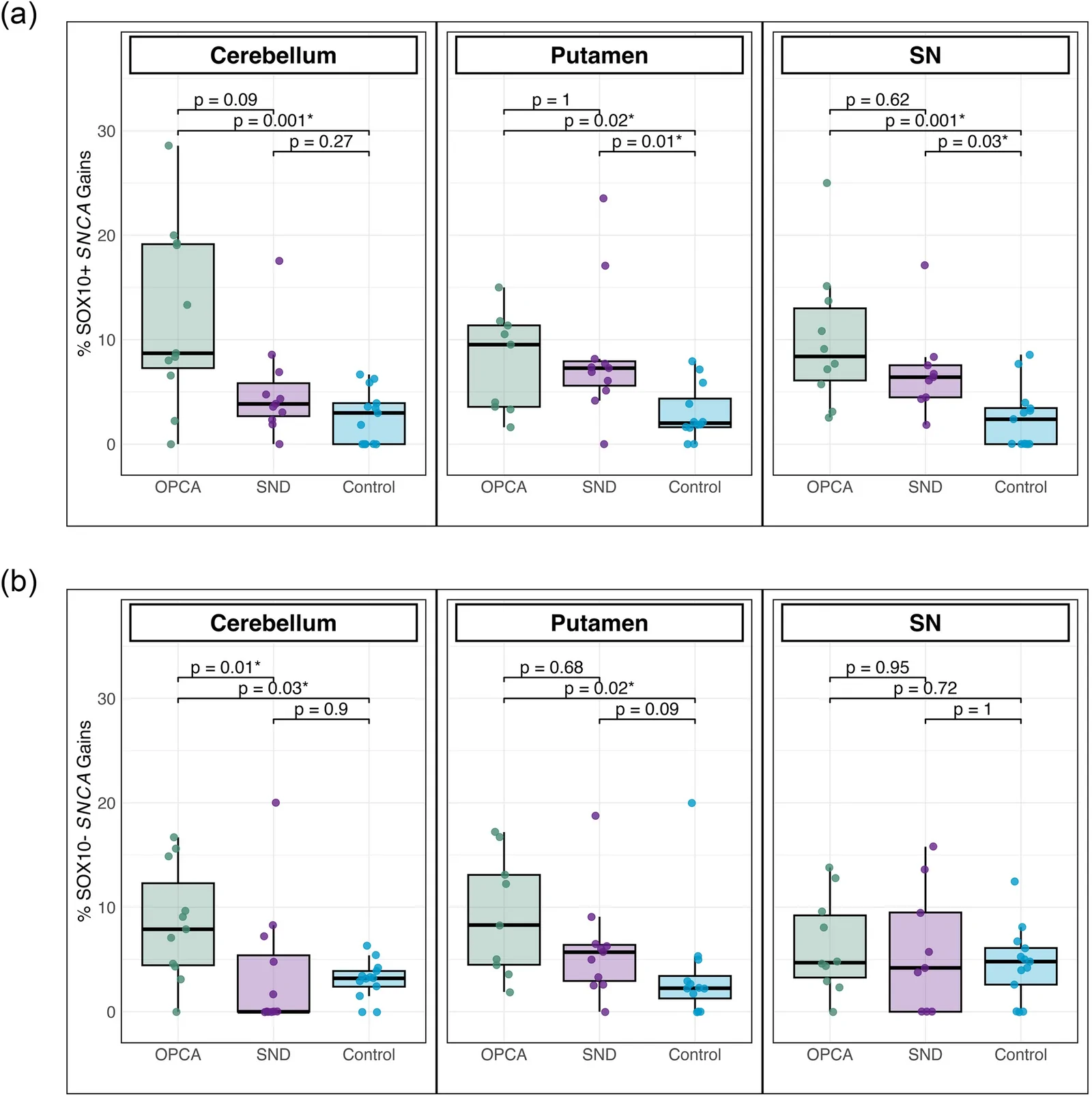
Distribution of percentage *SNCA* gains in (**a**) SOX10^+^ cells and (**b**) SOX10⁻ cells across the cerebellum, putamen and substantia nigra (SN). Regional panels compare three groups: OPCA, SND and control. Sample sizes (n) for each region and group are as follows: cerebellum—OPCA (*n* = 11), SND (*n* = 11), control (*n* = 13); putamen—OPCA (*n* = 9), SND (*n* = 11), control (*n* = 12); SN—OPCA (*n* = 10), SND (*n* = 9), control (*n* = 13). Statistical significance was assessed using the Kruskal–Wallis test followed by Dunn’s post hoc tests with Bonferroni correction for three comparisons. *p* values less than 0.05 are indicated by an asterisk (*)

Given the distinct regional patterns of pathology in MSA subtypes, with predominant involvement of the putamen in SND, the cerebellum in OPCA, and the substantia nigra in both, we next assessed whether the frequency of somatic *SNCA* gains in oligodendrocytes varies across these regions, as we had found in similar work without cell type characterisation [14] (Fig. 3a). In the OPCA subtype, significantly higher proportions of SOX10⁺ *SNCA* gains were observed compared to controls in all three regions examined: cerebellum (median 8.7% vs. 3.0%, adjusted* p* = 0.001), putamen (9.5% vs. 2.0%, adjusted* p* = 0.02) and substantia nigra (8.4% vs. 2.4%, adjusted* p* = 0.001), In the SND subtype compared to controls, significant increases were observed in the putamen (7.3% vs. 2.0%, adjusted* p* = 0.01) and substantia nigra (6.4% vs. 2.4%, adjusted* p* = 0.03), but not in the cerebellum (adjusted* p* = 0.27). When comparing *SNCA* oligodendrocyte gain frequencies in a given region between MSA subtypes, we noted a 2.2-fold higher difference in OPCA cerebellum compared to SND cerebellum (8.7% vs 3.9%); however, this was non-significant after correction (adjusted* p* = 0.09). We noted no difference between SND and OPCA putamen (7.3% vs 9.5%; adjusted* p* = 1). We next compared differentially affected regions within each MSA type. Within the SND group, we compared the putamen and cerebellum which revealed a 1.9-fold increase in SOX10⁺ gains in the putamen (7.3 vs 3.9%, adjusted* p* = 0.09). Within the OPCA group, there was no difference between the cerebellum and putamen (8.7% vs 9.5%, adjusted* p* = 0.40).

The relative enrichment of gains in the putamen compared to the cerebellum in SND, but lack of regional differences in SOX10⁺ *SNCA* gains in OPCA, could suggest that our OPCA cohort had a less pronounced cerebellar predominance, with the putamen affected almost to the same extent. To assess this, we compared the proportion of SOX10⁺ α-synuclein⁺ cells in preferentially affected regions (OPCA: cerebellum; SND: putamen) versus less affected regions (OPCA: putamen; SND: cerebellum) (Supplementary Fig. 3). In OPCA cases, the median percentage of SOX10⁺ inclusions was 26.1% in the cerebellum and 17.7% in the putamen, reflecting a 1.5-fold difference (*p* = 0.12). In contrast, SND cases exhibited a more pronounced 3.1-fold regional difference, with median values of 35.9% in the putamen and 11.5% in the cerebellum (*p* = 0.07). These findings support that our OPCA cohort has more regionally extensive pathology than SND, and therefore regional patterns of CNVs, if related to disease, would be expected primarily in SND.

Next, we performed the same analysis for SOX10⁻ cells (Fig. 3b). Significant differences in median percentages of gains were observed in the cerebellum and putamen of the OPCA group compared to controls (cerebellum: 7.9% vs. 3.2%, adjusted* p* = 0.03; putamen: 8.3% vs. 2.3%,* p* = 0.02). In the SND group, the difference between the putamen was not significant (5.7% vs. 2.3%, adjusted* p* = 0.09). A significant difference was observed in the cerebellum between OPCA and SND cases (7.9% vs 0%, adjusted *p* = 0.01), whereas no significant difference was found in the putamen. Interestingly, although the substantia nigra showed significant increases in SOX10^+^ cells, there were no group differences for SOX10⁻ gains. Within each MSA subtype, we noted no differences across any of the regions (OPCA: ANOVA *p* = 0.48; SND: Kruskal–Wallis *p* = 0.21). These results reflect a similar but less pronounced regional trend for *SNCA* gains in SOX10⁻ cells as there are for *SNCA* gains in oligodendrocytes.

Next, to determine whether *SNCA* gains in oligodendrocytes are associated with α-synuclein pathology at the single-cell level, we compared the proportion of SOX10^+^ oligodendrocytes containing inclusions between cells with a *SNCA* gain and those with copy number 2, stratified by brain region and MSA subtype (Table 2). This revealed that α-synuclein inclusions were present more than twice as often overall in oligodendrocytes with *SNCA* gains in the preferentially affected region of each subtype, compared to oligodendrocytes with normal CN in the same region (OPCA cerebellum, *p* = 0.00004, SND putamen. *p* = 0.00003) This was not the case in the less affected region in each MSA subtype (OPCA putamen, *p* = 0.91; SND cerebellum, *p* = 0.08). In the substantia nigra, OPCA had excess inclusions in oligodendrocytes with gains (*p* = 0.006), and a similar trend was seen in SND (*p* = 0.079). It should be noted, however, that the density of inclusions is lower in substantia nigra than the putamen or cerebellar white matter. These findings support the hypothesis that *SNCA* gains increase the susceptibility of individual oligodendrocytes to inclusion formation in predominantly affected regions. Consistent with this, the regional frequency of oligodendrocyte *SNCA* gains was significantly correlated with the overall burden of glial inclusions in the most affected regions of each MSA subtype (rho = 0.49,* p* = 0.02), supporting a role for *SNCA* gains in determining the regional distribution of pathology.

**Table 2:** Comparison of α-synuclein inclusions in cells with and without somatic *SNCA* gains

Region	MSA subtype	Donors (*n*)	Gains			CN2			OR	95% CI	*p* value
			Total	aSyn+	%	Total	aSyn+	%			
SOX10^+^ cells											
Cerebellum	OPCA	11	42	26	61.9	276	71	25.7	4.83	2.27–10.29	0.00004*
	SND	11	32	2	6.3	517	114	22.1	0.27	0.06 – 1.17	0.08
Putamen	OPCA	9	30	7	23.3	355	70	19.7	1.05	0.42–2.65	0.91
	SND	10	48	32	66.7	438	136	31.1	4.03	2.09–7.75	0.00003*
SN	OPCA	10	37	16	43.2	371	80	21.6	2.75	1.33 − 5.71	0.006*
	SND	8	30	9	30.0	360	59	16.4	2.13	0.92–4.94	0.079
SOX10⁻ cells											
Cerebellum	OPCA	11	47	7	14.9	439	18	4.1	4.13	1.44–11.80	0.008*
	SND	11	6	0	0	287	15	5.2	n/a	0—Inf	1
Putamen	OPCA	9	41	1	2.4	338	11	3.3	1.09	0.12—9.63	0.939
	SND	10	24	2	8.3	274	24	8.8	0.96	0.20 – 4.68	0.960
SN	OPCA	10	30	2	6.7	375	10	2.7	2.30	0.45—11.70	0.316
	SND	8	16	0	0	208	7	3.4	n/a	0 – inf	1

SOX10^+^ and SOX10⁻ cells shown separately. The OR estimates of the generalised linear mixed-effects model with 95% confidence intervals (CI) and corresponding *p* values are indicated. Statistically significant *p* values (< 0.05) are highlighted with an asterisk (*). Note that the OR does not apply to the SN and cerebellum in SOX10- cells, as none of the cells with gains had inclusions

Recently, evidence suggested that neuronal inclusions may also be a key driver of early MSA pathogenesis [71]. We therefore additionally quantified the relationship of *SNCA* gains and inclusions within SOX10⁻ cells (Table 2), with the limitation that SOX10⁻ cells comprise a heterogeneous mix of cell types, with inclusion-bearing cells likely neuronal, or SOX10⁻ oligodendrocytes, but more diverse amongst non-inclusion-bearing cells. This analysis revealed a significant excess (*p* = 0.008) for inclusions in SOX10⁻ cells with *SNCA* gains in the cerebellum of OPCA cases only. We cannot determine whether the inclusion-bearing cells were indeed neurons, which would indicate that gray matter was inadvertently included in the sample from which nuclear preparations were obtained, or oligodendrocytes not expressing detectable SOX10.

To examine this relationship from the converse perspective, we also summarised the distribution of *SNCA* copy number states amongst inclusion-positive and inclusion-negative cells (Supplementary Table 3). The majority of cells with inclusions had normal *SNCA* CN (71.2% for SOX10^+^, 68% for SOX10⁻). Reviewing the regional patterns in SOX10^+^ oligodendrocytes, we noted that *SNCA* gains were present almost four times more often overall in oligodendrocytes with inclusions in the preferentially affected region of each subtype, compared to oligodendrocytes without inclusions in the same region. This was not the case in the less affected region in each MSA subtype. In the substantia nigra, an almost twofold enrichment of gains was seen in oligodendrocytes with inclusions in both MSA subtypes. Thus, *SNCA* gains are not required for α-synuclein inclusion formation in a given oligodendrocyte, but they are associated with increased susceptibility to inclusion formation in specific affected regions.

### Somatic *SNCA* losses show widespread regional distributions and association with α-synuclein inclusions in the substantia nigra

**Fig. 4:**
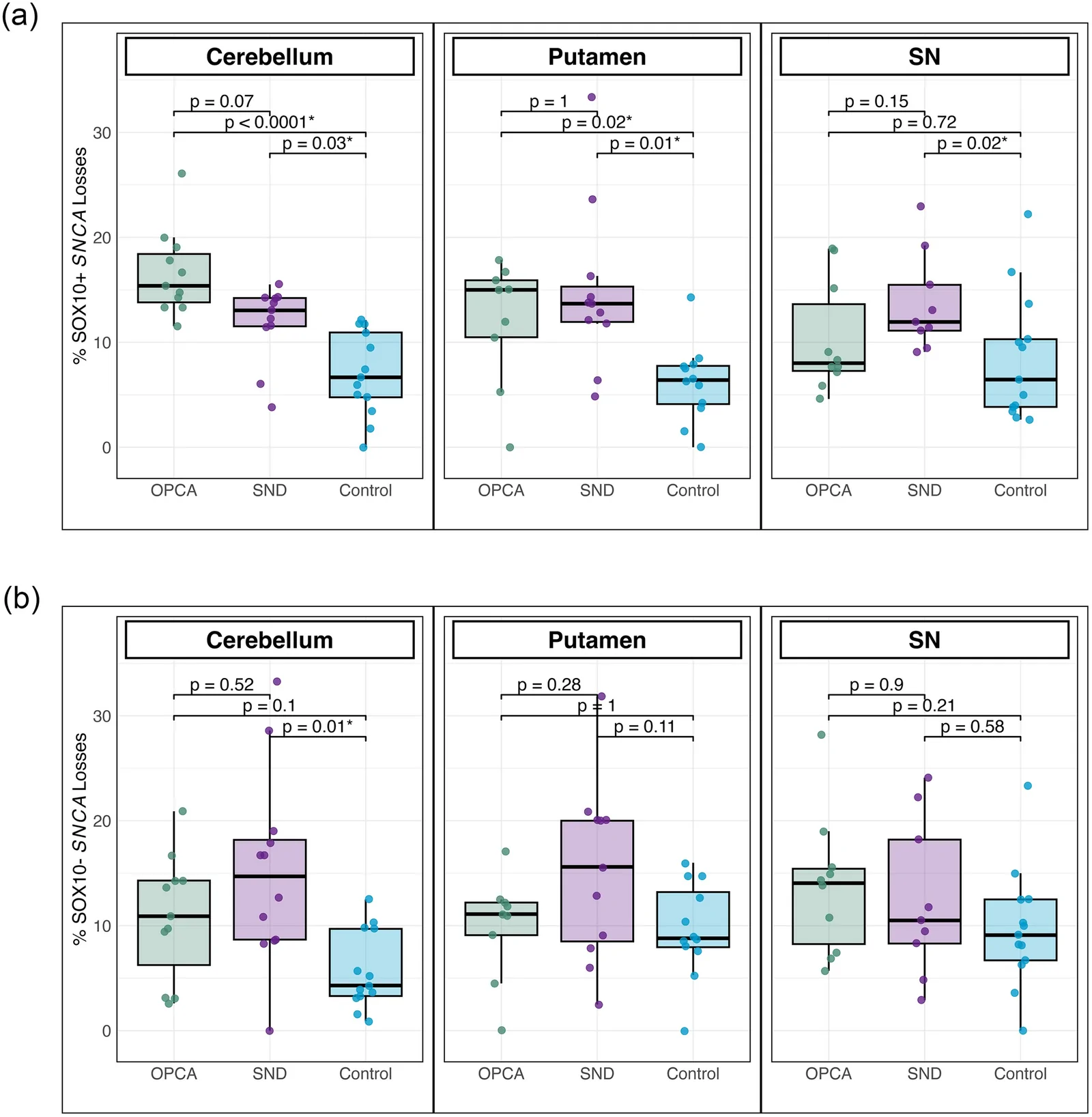
Distribution of percentage *SNCA* losses in (**a**) SOX10^+^ cells and (**b**) SOX10⁻ cells across the cerebellum, putamen and substantia nigra (SN). Regional panels compare three groups: OPCA, SND and control. Sample sizes (*n*) for each region and group are as follows: cerebellum—OPCA (*n* = 11), SND (*n* = 11), control (*n* = 13); putamen—OPCA (*n* = 9), SND (*n* = 11), control (*n* = 12); SN—OPCA (*n* = 10), SND (*n* = 9), control (*n* = 13). Statistical significance was assessed using the Kruskal–Wallis test followed by Dunn’s post hoc tests with Bonferroni correction for three comparisons. *p* values less than 0.05 are indicated by an asterisk (*)

Having established overall *SNCA* losses in MSA SOX10⁺ oligodendrocytes and SOX10⁻ cells, we next compared their levels across the cerebellum, putamen and substantia nigra amongst OPCA, SND and control groups (Fig. 4). In SOX10⁺ cells, *SNCA* losses were significantly more frequent in the cerebellum of OPCA cases (median 15.4%) and SND cases (13.0%) compared with controls (6.7%; adjusted* p* < 0.0001 and* p* = 0.03, respectively). In the putamen, both OPCA (15.0%) and SND (13.7%) showed increased *SNCA* losses versus controls (6.4%; adjusted* p* = 0.02 and* p* = 0.01, respectively). In the substantia nigra, we observed a significant difference between SND and control (11.9% vs 6.5%, adjusted* p* = 0.02) but not OPCA (8.0%,* p* = 0.72). Comparisons between SND and OPCA cases revealed no significant differences across any of the regions. Moreover, region-wise comparisons within each MSA subtype showed no significant differences (Kruskal–Wallis: OPCA *p* = 0.06; SND *p* = 0.77). For SOX10⁻ cells, *SNCA* losses were significantly elevated in the cerebellum of SND (14.7%) compared to controls (4.3%, adjusted* p* = 0.01), whereas the excess of OPCA losses was not significant (10.9%; adjusted *p* = 0.1). In the putamen, *SNCA* loss frequencies did not differ significantly between controls and either MSA subtype (controls: 8.8%; OPCA: 11.1%; SND: 15.6%). Similarly, the substantia nigra showed no significant group differences (SND: 10.5%; OPCA: 14.1%; controls: 9.1%). *SNCA* loss frequencies were also comparable across regions within each MSA subtype (Kruskal–Wallis: OPCA *p* = 0.36; SND *p* = 0.81). These results indicate that *SNCA* losses in MSA do not show a clear regional predilection based on differential regional involvement in each subtype.

As we had shown a relationship of *SNCA* gains with α-synuclein inclusions at both the single cell and regional level, we next assessed this for *SNCA* losses at the single-cell level. A significant relationship of *SNCA* losses with inclusions was present in oligodendrocytes in the substantia nigra of both MSA subtypes (SND *p* = 0.014, OPCA *p* = 0.0079), but not in other brain regions, although a similar trend was seen in SND putamen (*p* = 0.073), but not OPCA cerebellum (p = 0.421) (Table 3). In SOX10⁻ cells, there was a significant relationship of *SNCA* losses with inclusions only in OPCA substantia nigra (*p* = 0.014), with no clear effect in SND substantia nigra (*p* = 0.197), and a trend in OPCA putamen (*p* = 0.076) (Table 3). These results suggest that, unlike *SNCA* gains, the association of losses with inclusions in the same only cell occurs in substantia nigra oligodendrocytes in both MSA subtypes, and in other substantia nigra cells in OPCA only, but is marginal or absent in other regions.

**Table 3:** Comparison of α-synuclein inclusions in cells with and without somatic *SNCA* losses

Region	MSA subtype	Donors (n)	Losses			CN2			OR	95% CI	*p *value
			Total	aSyn+	%	Total	aSyn+	%			
SOX10^+^ cells											
Cerebellum	OPCA	11	62	18	29.0	276	71	25.7	1.3	0.68–2.48	0.421
	SND	11	77	21	27.3	517	114	22.1	1.33	0.74–2.37	0.338
Putamen	OPCA	9	58	14	24.1	355	70	19.7	1.20	0.61—2.37	0.593
	SND	10	72	31	43.1	438	136	31.1	1.63	0.96- 2.77	0.073
SN	OPCA	10	45	19	42.2	371	80	21.5	2.46	1.27—4.78	0.008*
	SND	8	60	19	31.7	360	59	16.4	2.18	1.18—4.06	0.013*
SOX10⁻ cells											
Cerebellum	OPCA	11	55	5	9.1	439	18	4.1	2.15	0.70—6.67	0.184
	SND	11	42	5	11.9	287	15	5.2	1.89	0.58—6.03	0.290
Putamen	OPCA	9	46	4	8.7	338	11	3.3	3.23	0.89—11.78	0.076
	SND	10	49	5	10.2	274	24	8.8	1.17	0.40—3.37	0.776
SN	OPCA	10	61	6	9.8	375	10	2.7	3.84	1.32—11.20	0.014*
	SND	8	35	3	8.6	208	7	3.4	2.50	0.62—10.11	0.197

The OR estimates of the generalised linear mixed-effects model with 95% confidence intervals (CI) and corresponding *p *values are indicated. Statistically significant *p *values (< 0.05) are highlighted with an asterisk (*)

### *SNCA* gains reflect an intrinsic case-level propensity for gains in other cell types and other brain regions associated with younger age of disease onset

**Fig. 5:**
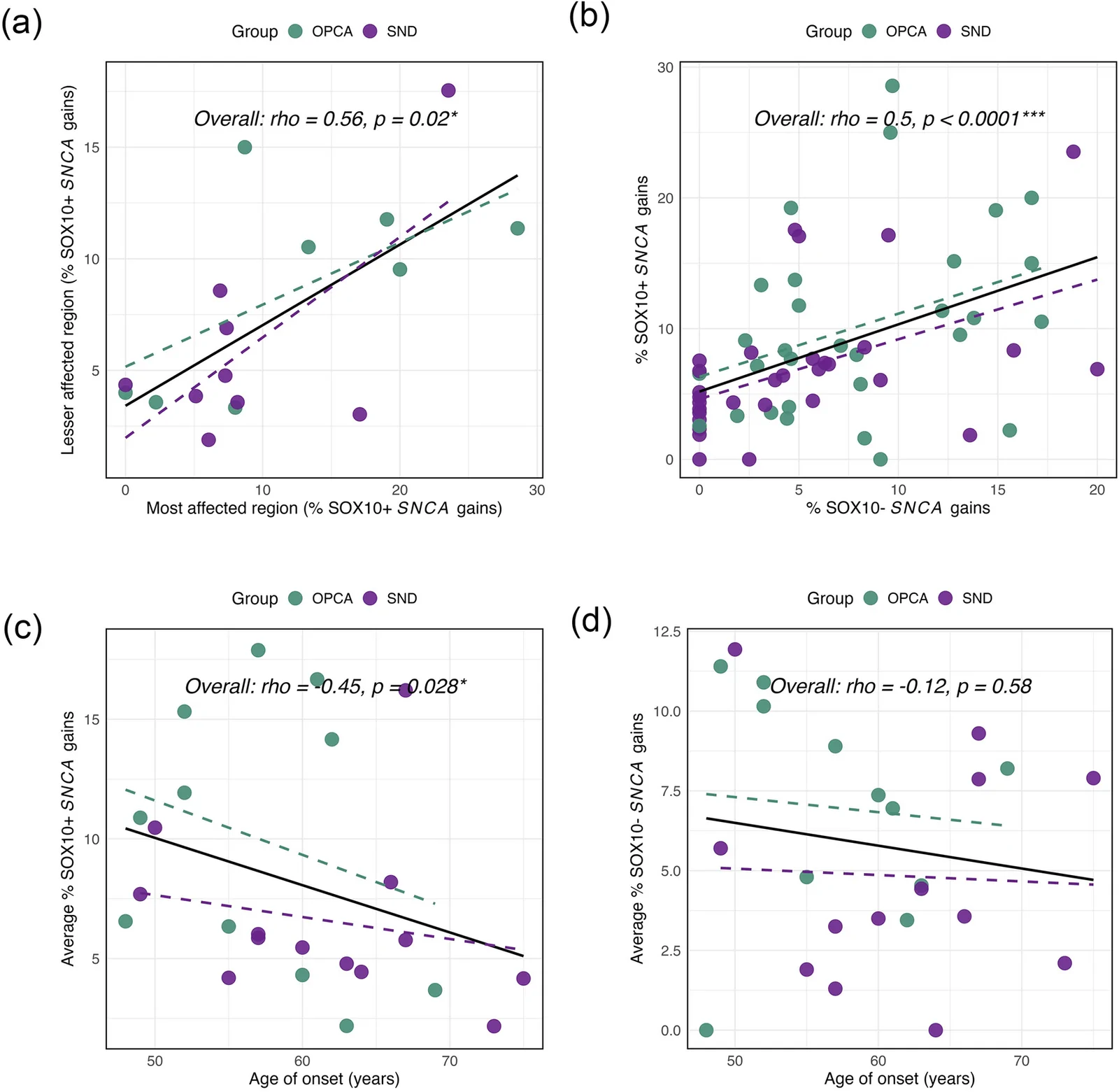
Spearman correlation analyses examining relationships between (**a**) percentage of SOX10^+^ *SNCA* gains in the region most affected in each subtype (OPCA cerebellum; SND putamen) compared with the region lesser affected (OPCA putamen; SND cerebellum) (OPCA, *n* = 8; SND, *n* = 9). (**b**) Percentage of SOX10^+^ *SNCA* gains plotted against SOX10⁻ *SNCA* gains across all regions analysed (OPCA, *n* = 30; SND, *n* = 31). (**c**) Average percentage of SOX10^+^ *SNCA* gains plotted against age at disease onset (OPCA, *n* = 11; SND, *n* = 13). (**d**) Average percentage of SOX10⁻ gains plotted against age at disease onset (OPCA, *n* = 11; SND, *n* = 13). Each panel includes a linear model illustrating the overall trend (black) together with separate trend lines for OPCA and SND

As we had seen apparent inter-individual variability in our CNV analysis, we explored whether this variability reflects a case-level propensity rather than being restricted to region-specific pathology. We first examined within-individual patterns of oligodendrocyte *SNCA* gains across brain regions by performing pairwise Spearman’s rank correlation analyses between the most and lesser affected brain regions within each case, revealing a significant positive correlation (rho = 0.56,* p* = 0.02; Fig. 5a). To determine whether cases with higher mosaicism in oligodendrocytes also had higher mosaicism in other cell types, we compared the frequency of *SNCA* gains in SOX10⁺ and SOX10⁻ cells across all regions, which also showed a significant positive correlation in MSA (rho = 0.5,* p* = < 0.0001) (Fig. 5b) but not in controls (rho = 0.2,* p* = 0.2). Taken together, this supports the idea that *SNCA* gains may represent an intrinsic, case-level tendency in MSA, across regions and cell types. We performed the same analysis for *SNCA* losses. No significant correlations were observed between SOX10⁺ and SOX10⁻ cells (rho = − 0.002,* p* = 1). Losses did, however, show a strong correlation between the most and lesser affected regions (rho = 0.7,* p* = 0.002).

Given the previous observation in PD of a negative correlation between age of onset and *SNCA* gains in the key affected region, the substantia nigra, and cell type (dopaminergic neurons) [43], we wondered whether such a relationship might exist for MSA. We investigated whether SOX10^+^ oligodendrocyte *SNCA* gains were associated with age of onset by taking the average percentage per case across the regions analysed. This revealed a significant negative correlation (rho = − 0.45,* p* = 0.03) for age of disease onset (Fig. 5c), which was not observed for SOX10⁻ *SNCA* gains (rho = − 0.12, *p* = 0.58) (Fig. 5d). There was no significant correlation with age of death (rho = − 0.34,* p* = 0.09), disease duration (rho = 0.10,* p* = 0.63) or PMI (rho = 0.27,* p* = 0.19). We did not detect any significant associations between *SNCA* losses in SOX10^+^ and SOX10⁻ cells and these clinical parameters (Supplementary Table 4). These results suggest that oligodendrocyte *SNCA* gains, but not *SNCA* gains or losses in SOX10⁻ cells, may have a role in determining onset age.

### DNA double-stranded breaks are enriched in MSA oligodendrocytes and are associated with α-synuclein pathology

**Fig. 6:**
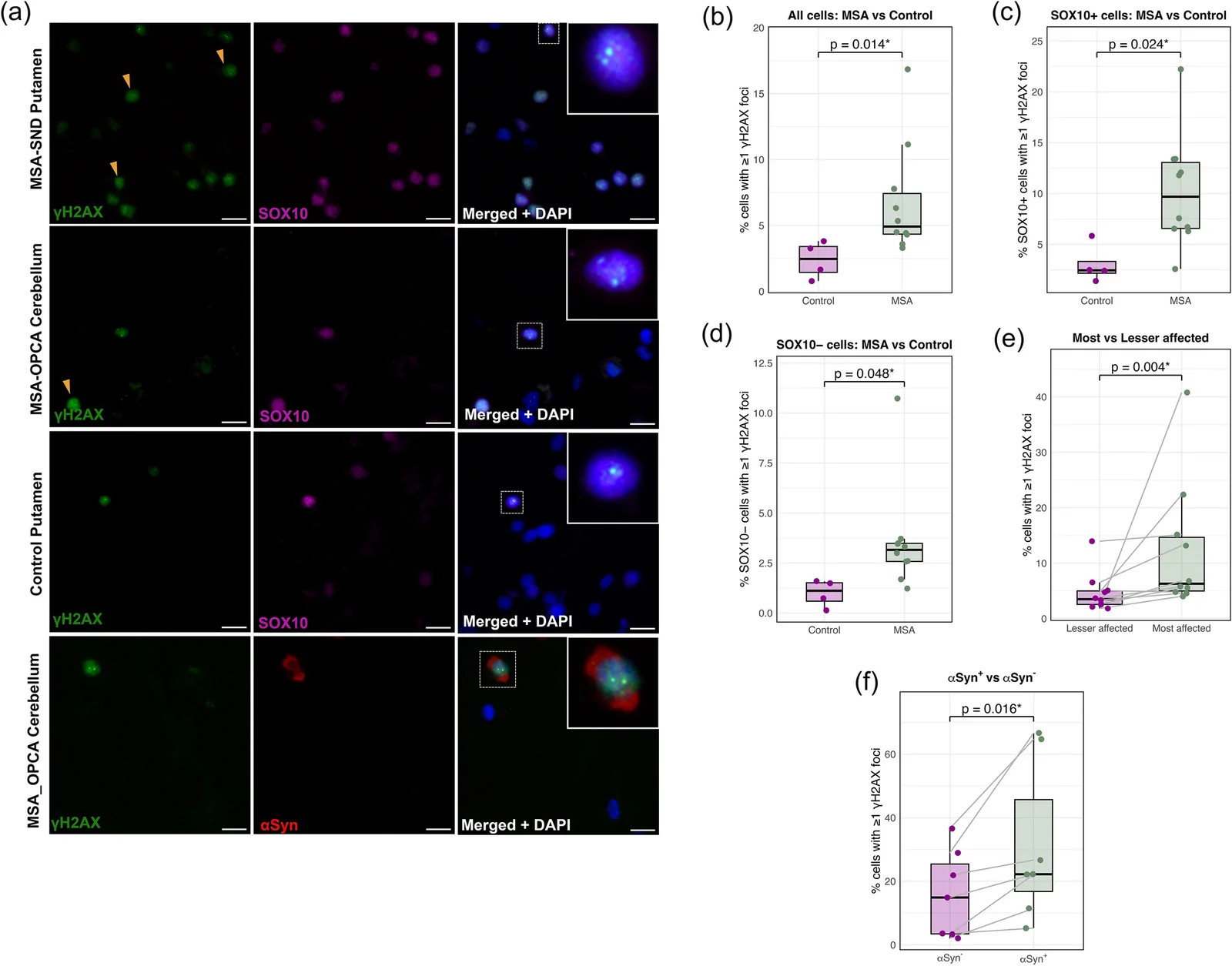
**a** Immunofluorescence images showing representative γH2AX staining. The upper three rows show γH2AX staining together with SOX10 staining in MSA-SND putamen, MSA-OPCA cerebellum and control putamen. The lower row shows γH2AX together with α-synuclein in MSA tissue. For each row, the individual markers are shown separately followed by a merged image that includes an inset with representative nuclei containing γH2AX staining. Orange arrows indicate examples of pan-nuclear γH2AX signal. Scale bar = 10 μm. **b** Quantification of cells that contain ≥ 1 γH2AX foci in MSA (*n* = 10) compared with control (*n* = 4). **c** Proportion of SOX10^+^ cells that contain ≥ 1 γH2AX labelling foci in MSA (*n* = 10) compared with control (*n* = 4). **d** Proportion of SOX10⁻ cells with ≥ 1 γH2AX positivity in MSA (*n* = 10) compared with control (*n* = 4). **e** Percentage of γH2AX positive cells in the region most affected for each subtype (OPCA cerebellum; SND putamen) compared with the region lesser affected (OPCA putamen; SND cerebellum) (*n* = 10). **f** Paired comparison of α-synuclein (aSyn)+ versus aSyn- cells with ≥ 1 γH2AX foci (*n* = 7). Panels b–d were analysed using linear regression with PMI as a covariate. Paired comparisons were performed using the Wilcoxon signed-rank test. Significant values <0.05 are indicated with an asterisk (*)

To investigate whether DSBs are associated with MSA pathology, we performed immunofluorescence staining for γH2AX phosphorylated at Ser139, an established marker of DSBs [37], in at least two regions from four controls, five SND cases and five OPCA cases (demographics summarised in Supplementary Table 5). The cerebellum and putamen were included in all brains and the substantia nigra where available (four MSA cases, three controls). Age at death was comparable between controls (median 64.5 years [IQR 57.5–71]) and MSA cases (median 64 years [IQR 58.8–67]) (p = 0.57). PMI was generally longer in controls, although this was not significant (median values: controls 79 h [IQR 66.7–87]; MSA 63.2 h [IQR 53.2–72.8]; Wilcoxon *p* = 0.44). Subsets of experiments included γH2AX with either SOX10 (all ten cases and four controls) or α-synuclein (six cases, and three controls where it was negative as expected) (Supplementary Table 6). Simultaneous detection of all three markers was not possible due to species overlap and secondary antibody compatibility. We observed two γH2AX staining patterns, diffuse pan-nuclear staining and bright foci (Fig. 6a). Pan-nuclear γH2AX signalling has been attributed to clustered DNA damage associated with several pathogenic cellular responses such as hypoxia, apoptosis and replication stress [12, 40, 42, 45], as well as more non-specific physiological responses such as increased cellular activity [57]. Accordingly, we focussed on analysing the discrete γH2AX foci, irrespective of pan-nuclear staining, as these are more reliable indicators of an active downstream DNA damage response in response to DSB. We thus classified cells with one or more foci as γH2AX-positive.

We first aimed to determine whether the DSB response is altered in MSA compared to controls, comparing the proportions of cells with γH2AX across MSA and control brain. Due to imperfect PMI matching, we considered the possibility of an association between PMI and γH2AX measurements proportions in a given donor, either overall (rho = 0.10, *p* = 0.72) or where cell types were identified (SOX10^+^ rho = − 0.11, *p* = 0.71, SOX10- rho = − 0.21, *p* = 0.46). Despite the lack of a significant correlation, we performed linear regression using PMI as a covariate. Across all brain regions analysed, the median percentage of γH2AX^+^ cells was significantly higher in MSA than in controls (4.9% vs 2.5%, *p* = 0.014) (Fig. 6b), indicating an increase in DNA damage response activity. To identify the affected cell populations, we analysed separately the experiments where SOX10 staining had been included. This revealed a significant enrichment of DSBs in oligodendrocytes in MSA versus controls (9.7% vs 2.5%, *p* = 0.024) (Fig. 6c) indicating that DSBs are present in cell types known to be vulnerable in MSA. We also noted a higher level of γH2AX^+^ in SOX10⁻ cells than in controls, which was just significant (3.2% vs 1.1%; *p* = 0.048) (Fig. 6d) This pattern suggests that the DSB response is enriched within MSA oligodendroglial populations, but also to some extent in other cell types, although we could not differentiate neurons from others. To further remove any effect of unknown factors, such as mode of death, on individual MSA cases, we compared the most and least affected regions for all, as we did for FISH. This revealed a clear excess in the most affected regions (medians 6.3% v 3.5%, *p* = 0.004) (Fig. 6e).

We finally assessed whether DSB accumulation was linked to α-synuclein pathology in six MSA cases (four OPCA, two SND) where we had data from at least one region. Paired comparisons revealed significantly more γH2AX foci in α-synuclein+ nuclei than α-synuclein– nuclei (22.2% vs 14.9%,* p* = 0.02) (Fig. 6f), suggesting a strong association between α-synuclein aggregation and DSB burden at the single-cell level. This finding is consistent with previous reports in LBD, where γH2AX accumulation and p53 activation occur in neurons with nuclear in α-synuclein [31]. Nevertheless, it is important to note that almost 78% of cells with inclusions do not have evidence of DSB.

## Discussion

In this study, we build upon growing evidence implicating somatic *SNCA* copy number gains in MSA by defining their cellular distribution, regional patterns and relationship to α-synuclein inclusions, analysing three key regions in MSA and controls. Unlike our previous work, which was performed on tissue sections, we have now used nuclear suspensions, which also allowed us to call losses, whilst retaining inclusions for correlations with CNVs in the same cell. It is important to note that the process of nuclear isolation removes, by definition, the majority of the extranuclear structures, although it has been long established that certain compartments can be retained [72], and recent work has demonstrated feasibility of sorting nuclei based on retained perinuclear tau inclusions [22]. It is not surprising, however, that the morphology of inclusions in our nuclear preparations differs from these of classical GCI, especially as they may be more easily degraded by pepsin when the cytoplasm is removed, even though we use a shorter exposure than in sections. Retained inclusions may, however, at least partly represent glial nuclear inclusions, although the latter are very rare, and typically occur in cells which also have GCIs [46]. The inability to specify inclusion type is a compromise needed in order to be able to call losses without sectioning artefacts.

We show that *SNCA* gains are increased in MSA across brain regions relative to controls, particularly in SOX10^+^ oligodendrocytes, the glial cell type predominantly affected by α-synuclein pathology. Within each subtype, oligodendrocyte *SNCA* gains tend to be high in regions that are selectively vulnerable, namely the cerebellum in OPCA and putamen in SND, and the substantia nigra in both. At the single-cell level, oligodendrocyte gains are associated with α-synuclein inclusions in affected regions, an association that was reflected at the regional level, where the frequency of gains correlated with the overall burden of α-synuclein pathology. These results, taken together with the association between higher oligodendrocyte *SNCA* gain burden and earlier age of disease onset, are supportive of a role in MSA pathogenesis, possibly by locally raising the intracellular α-synuclein to pathogenic thresholds. This is consistent with a recently reported strong association between cerebellar white matter pathology (overall, as well as GCI and number and density) and younger onset [28].

The regional distribution of oligodendrocyte *SNCA* gains differs between MSA subtypes. In SND, gains are enriched in the putamen relative to the cerebellum, consistent with the established regional predilection of pathology in this subtype. In contrast, in OPCA, oligodendrocyte *SNCA* gains are present at comparable levels in the cerebellum and putamen. In line with this, we found that the burden of GCIs in our cohort is 3-fold higher in SND in the putamen compared to the cerebellum, but only 1.5-fold higher in OPCA in the cerebellum compared to putamen. This may reflect the broader regional pathology seen in OPCA, where severe cerebellar and putaminal involvement often coexist [21], although we cannot exclude sampling bias or small sample size effects contributing to the pattern observed in our samples.

We also detected excess gains in SOX10⁻ cells consistent with our prior reports of neuronal *SNCA* mosaicism in MSA cingulate cortex and substantia nigra [43, 47] which may also contribute to disease pathogenesis. Neuronal inclusions are becoming more widely recognised as early events in MSA pathogenesis [71]. However, as we did not include neuronal markers in the present study, we cannot assign SOX10⁻ cells with inclusions to a neuronal lineage, as opposed to astrocytes, microglia, or indeed the possibility of some mature oligodendrocytes not having detectable SOX10 expression. Further experiments using neuronal and other cell type markers with α-synuclein would be necessary to assess this. In limited previous work in substantia nigra from seven MSA SND cases, the frequency of inclusions was 7.7% in dopaminergic neurons (detected by neuromelanin) with gains, against 6.1% in those without gains, whilst there was an apparent excess of inclusions in neuromelanin-negative cells with gains (33% vs 8%) [47].

Case-level analyses reveal that the level of *SNCA* gains in MSA is positively correlated across other brain regions and between SOX10^+^ oligodendrocytes and SOX10⁻ cells within the same individual, suggesting that these somatic gains reflect an intrinsic, case-specific propensity that is not observed in controls. These relationships may be consistent with an early clonal origin that produces descendant cells across multiple regions or cell types, supporting a model in which a subset of MSA patients may have pre-existing somatic *SNCA* mosaicism for gains that increases the likelihood of developing α-synuclein aggregation in later life. However, we cannot rule out the other possibility of a general increased susceptibility to *SNCA* copy number variation within MSA, in which case individual cells may carry unique rather than shared CNVs, with different size and distinct breakpoints. Whilst the former could be characterised fully by deep long read whole genome sequencing (WGS), the latter would require single-cell WGS. This is presently limited generally to megabase-scale CNVs [24] and could thus miss smaller events, although we did detect one in our pilot MSA single-cell WGS study [47]. Long read single-cell WGS could detect events of all sizes, and was recently reported in the brain in a small study including MSA [20].

Whilst these findings strengthen the case for a pathogenic role of somatic *SNCA* gains in MSA, our protocol on isolated nuclei also allowed the characterisation of somatic *SNCA* losses. Losses are also higher in MSA, and exhibit a more widespread distribution across cell types and regions. However, they showed a clear relationship to inclusions in the same cell only in the substantia nigra, and no correlation with onset age or disease duration, arguing against causality. This is not surprising because, unlike *SNCA* gains, germline losses have never, to our knowledge, been found in PD or MSA, and *SNCA*-null mice do not develop relevant phenotypes [1].

Collectively, these observations raise important questions regarding the timing and origin of these CNVs, which cannot be resolved with the current data. Gains and losses can arise through distinct mechanisms. Large-scale gains are most likely to reflect mitotic errors such as chromosome mis-segregation or DNA replication defects, and are typically clonal [5, 17]. This raises the likelihood that such gains occur prior to MSA pathogenesis, as the cells known to form α-synuclein inclusions are mostly post-mitotic, although we cannot exclude their emergence during low-level clonal expansion of glial progenitors in disease progression. We also cannot rule out that gains arise secondarily in post-mitotic cells, potentially through rare mechanisms such as cell-cycle re-entry and localised DNA synthesis, which has been reported in other neurodegenerative diseases [44, 55]. Importantly, gains and losses may sometimes occur concomitantly during non-allelic homologous recombination (NAHR), resulting in one daughter cell with a duplication and another with a reciprocal deletion [56], a process that could contribute to the mixed patterns of CNVs we observe. A somatic *SNCA* loss on the other hand could occur post-mitotically secondary to DNA damage in the form of DSBs, rather than a primary event. DSBs are the most severe form of DNA damage and can lead to widespread genomic deletions, which is particularly relevant in the brain as the main pathway for DSB repair in post-mitotic cells, NHEJ, is known to be error-prone [39, 51].

To explore this possibility, and to contextualise the *SNCA* CNV landscape within a broader framework of possible genomic instability, we explored DNA DSBs using γH2AX. We found widespread DNA DSB accumulation in MSA, more prominent overall in differentially affected regions. γH2AX foci were particularly enriched in SOX10 oligodendrocytes, but also in SOX10⁻ cells. They were also enriched in cells with α-synuclein inclusions, although we did not determine the cell types carrying the inclusions in these experiments, which may have included neurons. This finding suggests an association between α-synuclein aggregation and activation of DNA DSB repair in MSA. This is most likely in oligodendrocytes, but may also occur in neurons, as shown in Lewy body diseases [31, 63], although we cannot determine this in MSA, as we did not separate neurons from other SOX10⁻ cells. Importantly, more than three quarters of cells with inclusions did not have evidence of DSB, and further work will require cell type characterisation in parallel with DSB assessment to explore this further. We therefore hypothesise that somatic *SNCA* losses are secondary to disease progression, and may result from mis-repaired DSBs. To conclusively determine, however, whether *SNCA* losses result from DSBs would require combining FISH with γH2AX immunofluorescence. Our findings would also be strengthened by study of additional DNA damage response markers, such as 53BP1, and by a larger sample size. We have not specifically investigated apoptosis in our samples, although this is associated with a nuclear ring of γH2AX staining rather than discrete foci [61], and is therefore very unlikely to underlie our observations. Additionally, we cannot determine the timing of DNA DSB, although there was no significant correlation with PM interval.

This leads to the question of whether the propensity of the *SNCA* gene to acquire CNVs in MSA reflects specific instability of that gene, which has been suggested to be within a fragile site [54], or whether genome-wide somatic CNVs, known to occur in healthy brain cells [38], are enriched in MSA. The overall higher frequency of *SNCA* CNVs in controls relative to the reference probe suggests that the *SNCA* locus itself may be inherently prone to structural instability, independent of disease status, consistent with previous work showing minimal gains of other reference probes [14, 43]. The reference probe in our study was equally affected by CNVs (predominantly losses) in MSA and controls, arguing against an overall tendency for CNVs in MSA. Our pilot single-cell WGS of large CNVs genome-wide did detect them in ~30% of MSA cells, including a putaminal neuron with extensive nuclear inclusions and large-scale genomic losses encompassing *SNCA*, likely to have arisen by NHEJ [47]. Larger scWGS will be required, however, to determine if genome-wide CNVs are enriched in MSA.

Another limitation is that, whilst we propose sustained overexpression of *SNCA* mRNA as the mechanism by which gains contribute to pathogenesis, we have not demonstrated this. An increase in *SNCA* mRNA in MSA oligodendrocytes has been reported previously through quantitative reverse transcription PCR, though these results were non-significant [2]. More recently, a higher level of *SNCA* transcripts was found in oligodendrocytes with inclusions using RNAScope, though the numbers of cases analysed were limited [30]. It thus remains unclear whether *SNCA* mRNA is increased in MSA, especially across individual cell types. Ideally, methods assessing DNA and RNA in the same cell would clarify these relationships. Whilst in situ approaches are limited by DNA–RNA cross-reactivity, sequencing of the DNA and RNA of the same nucleus [67], although challenging, is becoming possible, including in the human brain [8]. Nonetheless, RNA measurements still only represent a snapshot at the point of death of cells surviving in a disease environment, rather than reflect long-term expression patterns, and are influenced by both pre-mortem and post-mortem changes [11, 65].

Whilst our findings strongly implicate somatic *SNCA* gains as contributors to MSA pathology, it is important to acknowledge that these gains represent only one facet of a multifactorial disease. Somatic mutations may serve as a “first hit” or modifier that increases vulnerability to α-synuclein aggregation. Supporting this, we did not find an association of *SNCA* gains with α-synuclein inclusions in the OPCA putamen, despite high levels of *SNCA* gains. Furthermore, both somatic *SNCA* gains and losses have been identified in neurologically healthy individuals from the UK Biobank data, though some of these had reported blood-based cancers, therefore it is unclear if they would be found also in the brain, and others were younger than the typical age of onset for MSA or PD [4]. Similarly, individuals with germline *SNCA* multiplications typically develop a clinical picture resembling PD rather than MSA, and do not always contain GCIs [27]. These observations demonstrate that there is no evidence for a simple pathogenic threshold, and somatic *SNCA* copy number variation alone cannot cause MSA without additional influences, such as cell-type-specific stress, environmental risk factors, coexisting inherited as yet unidentified genetic variants, or indeed other somatic mutations. Such mutations could arise within the brain or originate in the periphery, including in the context of clonal haematopoiesis of indeterminate potential, an age-related process recently reported to be associated with MSA [34]. At the single cell level, as the majority of the cells with inclusions did not have detectable *SNCA* gains, seeding from other cells may act as the trigger for inclusion formation without the requirement for a gain of *SNCA*, in addition to other pathogenic factors outlined above. Overall, our results point to somatic variation as a contributor to non-heritable genetic risk in MSA and helps advance our understanding of its aetiology. Remaining challenges include understanding why only some cells with gains develop inclusions, characterising SOX10⁻ cells with CNVs, and determining the temporal sequence by which gains and losses, and DNA DSBs, arise.

## Supplementary Information

Below is the link to the electronic supplementary material.

Supplementary file1 (PDF 2781 KB)

Supplementary file2 (XLSX 137 KB)

## Data Availability

All codes used to generate the statistical analyses and figures in this study are available on GitHub at https://github.com/caoimhemorley/somatic-SNCA-CNV-FISH-analysis. All raw cell counts and summarised datasets supporting the findings of this study are provided in the Supplementary Materials. Representative images are included in the figures, and additional raw image data are available from the corresponding author upon reasonable request.

## References

[1] Abeliovich A, Schmitz Y, Fariñas I, Choi-Lundberg D, Ho WH, Castillo PE et al (2000) Mice lacking α-synuclein display functional deficits in the nigrostriatal dopamine system. Neuron 25:239–252. 10.1016/S0896-6273(00)80886-7 10707987

[2] Asi YT, Simpson JE, Heath PR, Wharton SB, Lees AJ, Revesz T et al (2014) Alpha-synuclein mRNA expression in oligodendrocytes in MSA. Glia 62:964–970. 10.1002/glia.22653 24590631 PMC4238782

[3] Bendetowicz D, Fabbri M, Sirna F, Fernagut PO, Foubert-Samier A, Saulnier T et al (2024) Recent advances in clinical trials in multiple system atrophy. Curr Neurol Neurosci Rep 24:95–112. 10.1007/s11910-024-01335-0 38416311

[4] Blauwendraat C, Makarious MB, Leonard HL, Bandres-Ciga S, Iwaki H, Nalls MA et al (2021) A population scale analysis of rare SNCA variation in the UK Biobank. Neurobiol Dis 148:105182. 10.1016/j.nbd.2020.105182 33307186 PMC7880248

[5] Burssed B, Zamariolli M, Bellucco FT, Melaragno MI (2022) Mechanisms of structural chromosomal rearrangement formation. Mol Cytogenet 15:23. 10.1186/S13039-022-00600-6 35701783 PMC9199198

[6] Cabin DE, Shimazu K, Murphy D, Cole NB, Gottschalk W, McIlwain KL et al (2002) Synaptic vesicle depletion correlates with attenuated synaptic responses to prolonged repetitive stimulation in mice lacking alpha-synuclein. J Neurosci 22:8797–8807. 10.1523/JNEUROSCI.22-20-08797.2002 12388586 PMC6757677

[7] Chia R, Ray A, Shah Z, Ding J, Ruffo P, Fujita M et al (2024) Genome sequence analyses identify novel risk loci for multiple system atrophy. Neuron 112:2142-2156.e5. 10.1016/j.neuron.2024.04.002 38701790 PMC11223971

[8] Chung C, Yang X, Hevner RF, Kennedy K, Vong KI, Liu Y et al (2024) Cell-type-resolved mosaicism reveals clonal dynamics of the human forebrain. Nature 629:384–392. 10.1038/S41586-024-07292-5 38600385 PMC11194162

[9] Fanciulli A, Wenning GK (2015) Multiple-system atrophy. N Engl J Med 372:249–263. 10.1056/NEJMRA1311488 25587949

[10] Federoff M, Price TR, Sailer A, Scholz S, Hernandez D, Nicolas A et al (2016) Genome-wide estimate of the heritability of multiple system atrophy. Parkinsonism Relat Disord 22:35–41. 10.1016/j.parkreldis.2015.11.005 26589003 PMC4695377

[11] Ferreira PG, Muñoz-Aguirre M, Reverter F, Sá Godinho CP, Sousa A, Amadoz A et al (2018) The effects of death and post-mortem cold ischemia on human tissue transcriptomes. Nat Commun 9(1):9:490-. 10.1038/s41467-017-02772-x 29440659 PMC5811508

[12] Fragkos M, Choleza M, Papadopoulou P (2023) The role of γH2AX in replication stress-induced carcinogenesis: possible links and recent developments. Cancer Diagn Progn 3:639. 10.21873/CDP.10266 37927801 PMC10619570

[13] Gai WP, Pountney DL, Power JHT, Li QX, Culvenor JG, McLean CA et al (2003) α-synuclein fibrils constitute the central core of oligodendroglial inclusion filaments in multiple system atrophy. Exp Neurol 181:68–78. 10.1016/S0014-4886(03)00004-9 12710935

[14] Garcia-Segura ME, Perez-Rodriguez D, Chambers D, Jaunmuktane Z, Proukakis C (2023) Somatic SNCA copy number variants in multiple system atrophy are related to pathology and inclusions. Mov Disord 38:338–342. 10.1002/mds.29291 36448620

[15] Garcia-Segura ME, Perez-Rodriguez D, Proukakis C (2022) Combined fluorescent in situ hybridization (FISH) and immunofluorescence for the targeted detection of somatic copy number variants in synucleinopathies. Neuromethods. Springer Nature, pp 229–243

[16] Graham JG, Oppenheimer DR (1969) Orthostatic hypotension and nicotine sensitivity in a case of multiple system atrophy. J Neurol Neurosurg Psychiatry 32:28. 10.1136/JNNP.32.1.28 5774131 PMC493381

[17] Hastings PJ, Lupski JR, Rosenberg SM, Ira G (2009) Mechanisms of change in gene copy number. Nat Rev Genet 10(8):10:551-564. 10.1038/nrg2593 19597530 PMC2864001

[18] Huang H, He W, Tang T, Qiu M (2022) Immunological markers for central nervous system glia. Neurosci Bull 39(3):39:379-392. 10.1007/S12264-022-00938-2 36028641 PMC10043115

[19] Ikeuchi T, Kakita A, Shiga A, Kasuga K, Kaneko H, Tan CF et al (2008) Patients homozygous and heterozygous for SNCA duplication in a family with parkinsonism and dementia. Arch Neurol 65:514–519. 10.1001/ARCHNEUR.65.4.514 18413475

[20] Izydorczyk MB, Kalef-Ezra E, Horner DW, Zheng X, Holmes N, Toffoli M et al (2025) Single cell long read whole genome sequencing reveals somatic transposon activity in human brain. Commun Biol 8(1):8:1627-. 10.1038/s42003-025-08805-2 41266782 PMC12635067

[21] Jellinger KA, Seppi K, Wenning GK (2005) Grading of neuropathology in multiple system atrophy: proposal for a novel scale. Mov Disord 20:S29–S36. 10.1002/MDS.20537 16092088

[22] Jin B, Brown KSM, Smirnov DS, Naik SM, Kirkham SL, Hennessey EL et al (2025) Neurons accumulate disease-specific somatic genomic changes across tau pathologic states in Alzheimer’s disease. bioRxiv. 10.1101/2025.05.26.65615241573967

[23] Kalef-Ezra E, Perez-Rodriguez D, Proukakis C (2022) Manual isolation of nuclei from human brain using CellRaft device and single nucleus Whole Genome Amplification v1. 10.17504/PROTOCOLS.IO.KXYGXZJJOV8J/V1

[24] Kalef-Ezra E, Turan ZG, Perez-Rodriguez D, Bomann I, Behera S, Morley C et al (2024) Single-cell somatic copy number variants in brain using different amplification methods and reference genomes. Commun Biol 7:1–10. 10.1038/S42003-024-06940-W;SUBJMETA 39384904 PMC11464624

[25] Kato S, Nakamura H (1990) Cytoplasmic argyrophilic inclusions in neurons of pontine nuclei in patients with olivopontocerebellar atrophy: immunohistochemical and ultrastructural studies. Acta Neuropathol 79:584–594. 10.1007/BF00294235 2163181

[26] Khodr CE, Sapru MK, Pedapati J, Han Y, West NC, Kells AP et al (2011) An alpha-synuclein AAV gene silencing vector ameliorates a behavioral deficit in a rat model of Parkinson’s disease, but displays toxicity in dopamine neurons. Brain Res 1395:94. 10.1016/J.BRAINRES.2011.04.036 21565333 PMC3105182

[27] Kiely AP, Ling H, Asi YT, Kara E, Proukakis C, Schapira AH et al (2015) Distinct clinical and neuropathological features of G51D SNCA mutation cases compared with SNCA duplication and H50Q mutation. Mol Neurodegener 10:1–17. 10.1186/S13024-015-0038-3 26306801 PMC4549856

[28] Kim A, Yoshida K, Kovacs GG, Forrest SL (2024) Computer-based evaluation of α-synuclein pathology in multiple system atrophy as a novel tool to recognize disease subtypes. Mod Pathol 37:100533. 10.1016/J.MODPAT.2024.100533 38852813

[29] Koga S, Sekiya H, Kondru N, Ross OA, Dickson DW (2021) Neuropathology and molecular diagnosis of Synucleinopathies. Mol Neurodegener 16:1–24. 10.1186/S13024-021-00501-Z/TABLES/3 34922583 PMC8684287

[30] Kon T, Forrest SL, Lee S, Li J, Chasiotis H, Nassir N et al (2024) SNCA and TPPP transcripts increase in oligodendroglial cytoplasmic inclusions in multiple system atrophy. Neurobiol Dis 198:106551. 10.1016/J.NBD.2024.106551 38839023

[31] Koss DJ, Todd O, Menon H, Anderson Z, Yang T, Findlay L et al (2025) A reciprocal relationship between markers of genomic DNA damage and alpha-synuclein pathology in dementia with Lewy bodies. Mol Neurodegener 20(1):20–25. 10.1186/S13024-025-00813-4 40114198 PMC11927131

[32] Krismer F, Fanciulli A, Meissner WG, Coon EA, Wenning GK (2024) Multiple system atrophy: advances in pathophysiology, diagnosis, and treatment. Lancet Neurol 23:1252–1266. 10.1016/S1474-4422(24)00396-X 39577925

[33] Lázaro DF, Lee VMY (2024) Navigating through the complexities of synucleinopathies: insights into pathogenesis, heterogeneity, and future perspectives. Neuron 112:3029–3042. 10.1016/J.NEURON.2024.05.017 38861985 PMC11427175

[34] Lee S, Kim HJ, Kim S, Jin B, Jeon HY, Woo KA et al (2024) Clonal hematopoiesis with DNMT3A mutations is associated with multiple system atrophy. Parkinsonism Relat Disord 128:107145. 10.1016/J.PARKRELDIS.2024.107145 39278121

[35] Leija-Salazar M, Piette C, Proukakis C (2018) Review: somatic mutations in neurodegeneration. Neuropathol Appl Neurobiol 44:267–285. 10.1111/nan.12465 29369391

[36] Lieber MR (2010) The mechanism of double-strand DNA break repair by the nonhomologous DNA end joining pathway. Annu Rev Biochem 79:181. 10.1146/ANNUREV.BIOCHEM.052308.093131 20192759 PMC3079308

[37] Mah LJ, El-Osta A, Karagiannis TC (2010) γH2AX: a sensitive molecular marker of DNA damage and repair. Leukemia 24(4):679–686. 10.1038/leu.2010.6 20130602

[38] McConnell MJ, Lindberg MR, Brennand KJ, Piper JC, Voet T, Cowing-Zitron C et al (2013) Mosaic copy number variation in human neurons. Science 342:632–637. 10.1126/SCIENCE.1243472 24179226 PMC3975283

[39] McKinnon PJ (2009) DNA repair deficiency and neurological disease. Nat Rev Neurosci 10:100. 10.1038/NRN2559 19145234 PMC3064843

[40] Meyer B, Voss KO, Tobias F, Jakob B, Durante M, Taucher-Scholz G (2013) Clustered DNA damage induces pan-nuclear H2AX phosphorylation mediated by ATM and DNA–PK. Nucleic Acids Res 41:6109. 10.1093/NAR/GKT304 23620287 PMC3695524

[41] Miller MB, Huang AY, Kim J, Zhou Z, Kirkham SL, Maury EA et al (2022) Somatic genomic changes in single Alzheimer’s disease neurons. Nature 604:714–722. 10.1038/s41586-022-04640-1 35444284 PMC9357465

[42] Moeglin E, Desplancq D, Conic S, Oulad-Abdelghani M, Stoessel A, Chiper M et al (2019) Uniform widespread nuclear phosphorylation of histone H2AX is an indicator of lethal DNA replication stress. Cancers. 10.3390/CANCERS11030355 30871194 PMC6468890

[43] Mokretar K, Pease D, Taanman J-W, Soenmez A, Ejaz A, Lashley T et al (2018) Somatic copy number gains of α-synuclein (SNCA) in Parkinson’s disease and multiple system atrophy brains. Brain 141:2419–2431. 10.1093/brain/awy157 29917054

[44] Nandakumar S, Rozich E, Buttitta L (2021) Cell cycle re-entry in the nervous system: from polyploidy to neurodegeneration. Front Cell Dev Biol 9:698661. 10.3389/FCELL.2021.698661 34249947 PMC8264763

[45] Olcina MM, Grand RJA, Hammond EM (2014) ATM activation in hypoxia - causes and consequences. Mol Cell Oncol 1:e29903. 10.4161/MCO.29903 27308313 PMC4905164

[46] Papp MI, Lantos PL (1992) Accumulation of tubular structures in oligodendroglial and neuronal cells as the basic alteration in multiple system atrophy. J Neurol Sci 107:172–182. 10.1016/0022-510X(92)90286-T 1314292

[47] Perez-Rodriguez D, Kalyva M, Leija-Salazar M, Lashley T, Tarabichi M, Chelban V et al (2019) Investigation of somatic CNVs in brains of synucleinopathy cases using targeted SNCA analysis and single cell sequencing. Acta Neuropathol Commun 7:219. 10.1186/s40478-019-0873-5 31870437 PMC6929293

[48] Perez-Rodriguez D, Kalyva M, Santucci C, Proukakis C (2023) Somatic CNV Detection by Single-Cell Whole-Genome Sequencing in Postmortem Human Brain. pp 205–230 10.1007/978-1-0716-2655-9_11 36399272

[49] Pozniak CD, Langseth AJ, Dijkgraaf GJP, Choe Y, Werb Z, Pleasure SJ (2010) Sox10 directs neural stem cells toward the oligodendrocyte lineage by decreasing suppressor of fused expression. Proc Natl Acad Sci U S A 107:21795–21800. 10.1073/PNAS.1016485107 21098272 PMC3003047

[50] Proukakis C (2020) Somatic mutations in neurodegeneration: an update. Neurobiol Dis 144:105021. 10.1016/j.nbd.2020.105021 32712267

[51] Provasek VE, Mitra J, Malojirao VH, Hegde ML (2022) DNA double-strand breaks as pathogenic lesions in neurological disorders. Int J Mol Sci 23:4653. 10.3390/IJMS23094653 35563044 PMC9099445

[52] Robertson DC, Schmidt O, Ninkina N, Jones PA, Sharkey J, Buchman VL (2004) Developmental loss and resistance to MPTP toxicity of dopaminergic neurones in substantia nigra pars compacta of γ-synuclein, α-synuclein and double α/γ-synuclein null mutant mice. J Neurochem 89:1126–1136. 10.1111/j.1471-4159.2004.02378.x 15147505

[53] Rodgers K, Mcvey M (2016) Error-prone repair of DNA double-strand breaks. J Cell Physiol 231:15. 10.1002/JCP.25053 26033759 PMC4586358

[54] Rozier L, El-Achkar E, Apiou F, Debatisse M (2004) Characterization of a conserved aphidicolin-sensitive common fragile site at human 4q22 and mouse 6C1: Possible association with an inherited disease and cancer. Oncogene 23:6872–6880. 10.1038/SJ.ONC.1207809;KWRD 15286716

[55] Sah E, Krishnamurthy S, Ahmidouch MY, Gillispie GJ, Milligan C, Orr ME (2021) The Cellular Senescence Stress Response in Post-Mitotic Brain Cells: Cell Survival at the Expense of Tissue Degeneration 11:229. 10.3390/LIFE11030229 PMC7998276 33799628

[56] Sasaki M, Lange J, Keeney S (2010) Genome destabilization by homologous recombination in the germline. Nat Rev Mol Cell Biol 11:182. 10.1038/NRM2849 20164840 PMC3073813

[57] Shanbhag NM, Evans MD, Mao W, Nana AL, Seeley WW, Adame A et al (2019) Early neuronal accumulation of DNA double strand breaks in Alzheimer’s disease. Acta Neuropathol Commun. 10.1186/S40478-019-0723-5 31101070 PMC6524256

[58] Sharma M, Burré J (2022) α-Synuclein in synaptic function and dysfunction. Trends Neurosci 46:153. 10.1016/J.TINS.2022.11.007 36567199 PMC9877183

[59] Shults CW, Rockenstein E, Crews L, Adame A, Mante M, Larrea G et al (2005) Neurological and neurodegenerative alterations in a transgenic mouse model expressing human α-synuclein under oligodendrocyte promoter: implications for multiple system atrophy. J Neurosci 25:10689. 10.1523/JNEUROSCI.3527-05.2005 16291942 PMC6725840

[60] Sock E, Wegner M (2021) Using the lineage determinants Olig2 and Sox10 to explore transcriptional regulation of oligodendrocyte development. Dev Neurobiol 81:892–901. 10.1002/DNEU.22849 34480425

[61] Solier S, Pommier Y (2009) The apoptotic ring: a novel entity with phosphorylated histones H2AX and H2B, and activated DNA damage response kinases. Cell Cycle 8:1853–1859. 10.4161/CC.8.12.8865 19448405

[62] Spillantini MG, Schmidt ML, Lee VMY, Trojanowski JQ, Jakes R, Goedert M (1997) α-synuclein in Lewy bodies [8]. Nature 388:839–840. 10.1038/42166;KWRD 9278044

[63] Sproviero D, Payán-Gómez C, Milanese C, Barnhoorn S, Sun S, Gyenis A et al (2025) A blood-based DNA damage signature in patients with Parkinson’s disease is associated with disease progression. Nat Aging 5:1844–1861. 10.1038/S43587-025-00926-X 40913219 PMC12443628

[64] Stefanova N, Wenning GK (2023) Multiple system atrophy: at the crossroads of cellular, molecular and genetic mechanisms. Nat Rev Neurosci 24(6):24–346. 10.1038/s41583-023-00697-7 37085728

[65] Tomita H, Vawter MP, Walsh DM, Evans SJ, Choudary PV, Li J et al (2004) Effect of agonal and postmortem factors on gene expression profile: quality control in microarray analyses of postmortem human brain. Biol Psychiatry 55:346–352. 10.1016/J.BIOPSYCH.2003.10.013 14960286 PMC3098566

[66] Tu PH, Galvin JE, Baba M, Giasson B, Tomita T, Leight S et al (1998) Glial cytoplasmic inclusions in white matter oligodendrocytes of multiple system atrophy brains contain insoluble alpha-synuclein. Ann Neurol 44:415–422. 10.1002/ANA.410440324 9749615

[67] Vandereyken K, Sifrim A, Thienpont B, Voet T (2023) Methods and applications for single-cell and spatial multi-omics. Nat Rev Genet 24(8):24–515. 10.1038/s41576-023-00580-2 PMC9979144 36864178

[68] Verheijen BM, Vermulst M, van Leeuwen FW (2018) Somatic mutations in neurons during aging and neurodegeneration. Acta Neuropathol 135:811. 10.1007/S00401-018-1850-Y 29705908 PMC5954077

[69] Volpicelli-Daley LA, Luk KC, Patel TP, Tanik SA, Riddle DM, Stieber A et al (2011) Exogenous α-synuclein fibrils induce Lewy body pathology leading to synaptic dysfunction and neuron death. Neuron 72:57–71. 10.1016/J.NEURON.2011.08.033 21982369 PMC3204802

[70] Williams GP, Marmion DJ, Schonhoff AM, Jurkuvenaite A, Won WJ, Standaert DG et al (2020) T cell infiltration in both human multiple system atrophy and a novel mouse model of the disease. Acta Neuropathol 139:855–874. 10.1007/S00401-020-02126-W 31993745 PMC7181566

[71] Wiseman JA, Halliday GM, Dieriks BV (2025) Neuronal α-synuclein toxicity is the key driver of neurodegeneration in multiple system atrophy. Brain 148:2306. 10.1093/BRAIN/AWAF030 39908177 PMC12233558

[72] Xu X, Stoyanova EI, Lemiesz AE, Xing J, Mash DC, Heintz N (2018) Species and cell-type properties of classically defined human and rodent neurons and glia. Elife. 10.7554/ELIFE.37551 30320555 PMC6188473

[73] Zafar F, Valappil RA, Kim S, Johansen KK, Chang ALS, Tetrud JW et al (2018) Genetic fine-mapping of the Iowan *SNCA* gene triplication in a patient with Parkinson’s disease. NPJ Parkinsons Dis 4:18. 10.1038/S41531-018-0054-4 29928688 PMC6003950

