# Supplementary material for: Oligodendroglial somatic SNCA copy number gains are associated with inclusions and disease onset in multiple system atrophy
Source: Acta Neuropathol. 2026 Sep 4;152(1):31. doi: 10.1007/s00401-026-03077-4 (PMC13545049; doi:10.1007/s00401-026-03077-4)
Supplement: Supplementary file 1 [file 401_2026_3077_MOESM1_ESM.pdf]

Supplementary Table 1. Summary of case demographics and experiments performed

			Age of onset (years)	Age of death (years)	PMI (hours)	One-colour FISH (*SNCA*, SOX10, aSyn)			Two-colour FISH (*SNCA*, Chr 7 CEP, SOX10)			γH2AX immunostaining		
**Donor ID**	**Sex**	**Diagnosis**				**CER**	**SN**	**PUT**	**CER**	**SN**	**PUT**	**CER**	**SN**	**PUT**
SND01	F	SND	64	70	34.0	X	X	X						
SND02	F	SND	67	75	36.8	X	X	X						
SND03	F	SND	57	64	57.0							X	X	
SND04	F	SND	66	71	53.0	X	X	X						
SND05	M	SND	57	61	62.3	X		X	X		X			
SND06	M	SND	75	82	41.0	X	X							
SND07	F	SND	49	55	45.0			X						
SND08	M	SND	67	72	107.0	X	X	X						
SND09	F	SND	55	63	28.7	X	X							
SND10	M	SND	73	80	43.8	X		X						
SND11	F	SND	55	67	73.5								X	
SND12	F	SND	60	67	28.0					X	X	X		
SND13	M	SND	57	64	49.6	X		X						
SND14	M	SND	50	56	39.0	X	X	X						
SND15	F	SND	63	67	79.0	X	X	X						
Total						10	8	10	1	1	2	2	2	0
OPCA01	M	OPCA	57	67	81.2	X	X	X						
OPCA02	M	OPCA	63	70	64.1	X	X	X				X		
OPCA03	F	OPCA	49	63	46.3	X	X	X						
OPCA04	F	OPCA	52	58	70.8	X	X	X					X	X
OPCA05	M	OPCA	48	54	88.1	X						X		
OPCA06	F	OPCA	62	69	62.7	X	X							
OPCA07	F	OPCA	60	66	60.5	X	X	X						

OPCA08	F	OPCA	61	71	28.7	X	X							
OPCA09	M	OPCA	69	73	85.3		X	X						
OPCA10	M	OPCA	55	68	103.0	X	X	X				X	X	
OPCA11	F	OPCA	52	57	88.4	X		X						
OPCA12	F	OPCA	N/A	54	52.0	X	X	X		X	X	X		
Total						11	10	9	0	1	1	4	2	1
Control01	M	Control	N/A	69	168.0		X	X	X					
Control02	M	Control	N/A	73	47.0		X				X			
Control03	F	Control	N/A	57	N/A				X	X				
Control04	M	Control	N/A	74	101.3				X		X			
Control05	F	Control	N/A	88	11.1				X	X	X			
Control06	M	Control	N/A	90	46.3	X				X	X			
Control07	F	Control	N/A	68	44.6				X	X	X			
Control08	M	Control	N/A	88	16.2	X	X	X						
Control09	F	Control (dystonia)	N/A	58	102.0				X	X	X			
Control10	M	Control	N/A	58	78.5					X		X	X	X
Control11	M	Control	N/A	71	38.5			X	X	X				
Control12	F	Control	N/A	56	82.0				X			X	X	X
Control13	F	Control	N/A	73	24.0				X	X	X			
Control14	F	Control	N/A	71	76.1				X	X	X	X	X	
Control15	M	Control	N/A	76	39.1	X				X	X			
Total						3	3	3	10	10	9	3	3	2

Demographic information (sex, diagnosis, age of onset, age of death, and post-mortem interval [PMI]) is shown for all donors included in the study. The table also indicates the brain regions analysed for each experimental approach. Brain regions analysed include cerebellum (CER), substantia nigra (SN), and putamen (PUT). N/A indicates data not available.

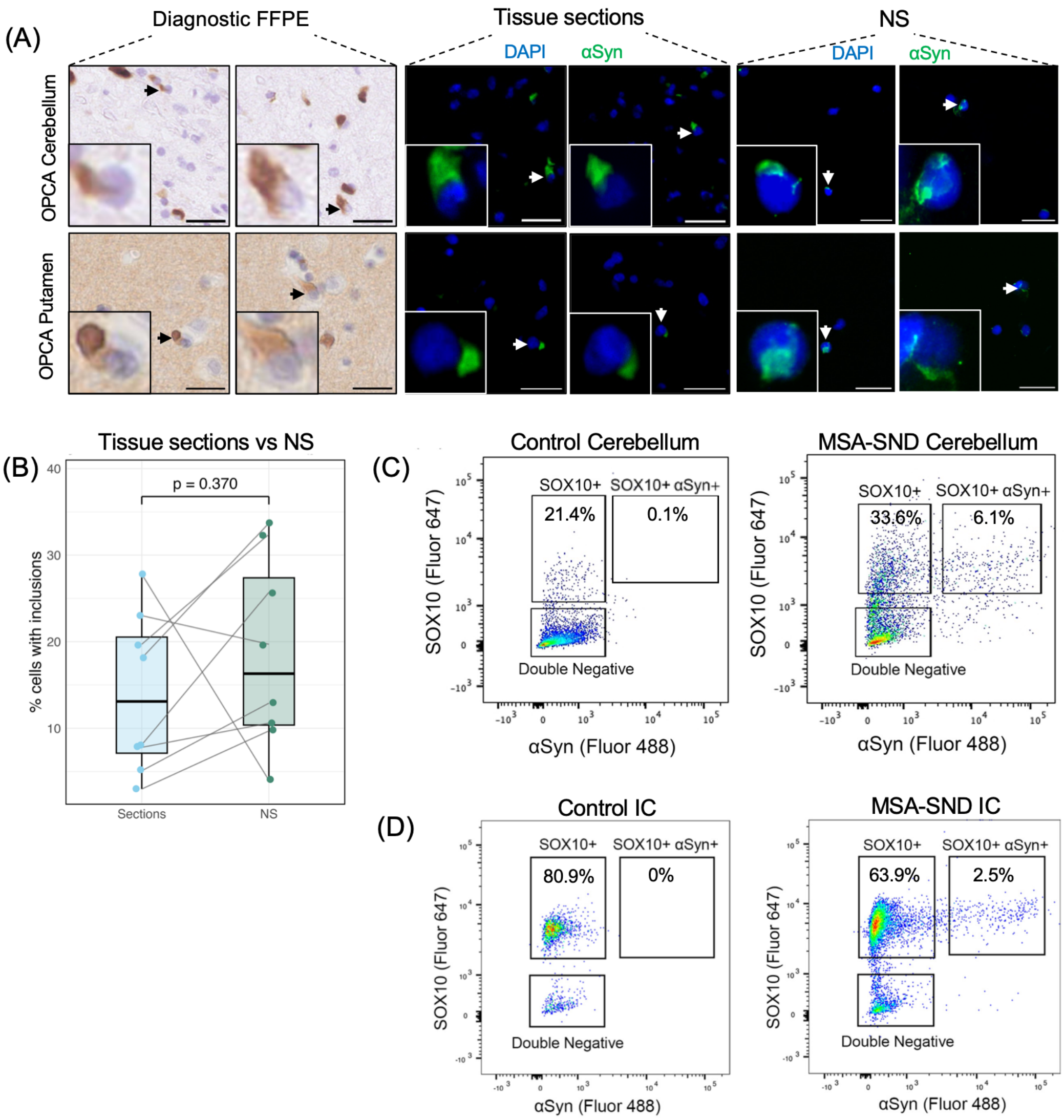

Supplementary Fig 1. Validation of α-synuclein inclusion detection following nuclei isolation (A) Representative images from two MSA-OPCA donors (upper and lower rows) demonstrating α-synuclein pathology in the cerebellum (OPCA05) and putamen (OPCA11) across three experimental preparations. Left: diagnostic formalin-fixed paraffin-embedded (FFPE) sections stained for α-synuclein by immunohistochemistry. Middle: frozen tissue sections immunolabelled for α-synuclein. Right: isolated nuclear suspensions immunolabelled for α-synuclein with DAPI nuclear counterstaining. Insets show representative α-synuclein inclusions. Scale bar = 20 μm. (B) Paired comparison of the percentage of α-synuclein-positive oligodendrocytes detected in frozen tissue sections and corresponding nuclear suspensions from the same case. Statistical significance was assessed using a paired t-test. (C) Flow cytometry gating strategy SOX10-positive and α-synuclein-positive nuclei from the cerebellum of a control donor (Control10) and an MSA-SND (SND12) donor. (D) Flow cytometry gating strategy for SOX10-positive and α-synuclein-positive isolated nuclei from the internal capsule (IC) of a control donor (Control11) and MSA-SND donor (SND14).

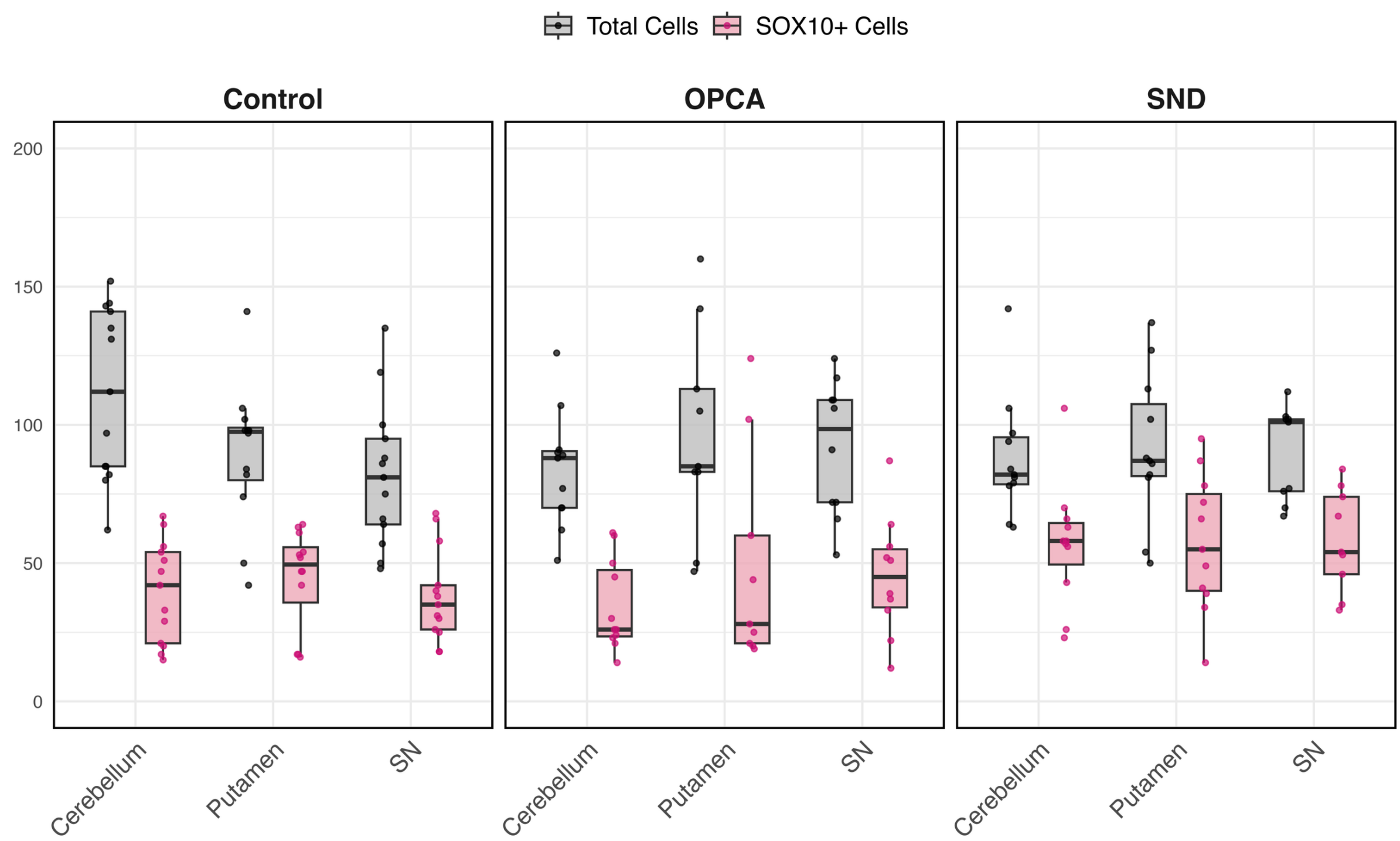

Supplementary Fig 2. Distribution of overall cell counts and SOX10+ cells across each group and region.

Supplementary Table 2. Summary of cell counts and *SNCA* CNVs per group and region

		Cells analysed				Gains				Losses			
Group	Region	Cell type	Total	Per case mean (SD)	Per case median [IQR]	Number	%	Median [IQR]	Mean (SD)	Number	%	Median [IQR]	Mean (SD)
Control	Cerebellum	SOX10+	516	39.7 (18.3)	42 [21–54]	14	2.7	3.0 [0–3.9]	2.7 (2.6)	35	6.8	6.7 [4.8–10.9]	7.0 (4.0)
		SOX10-	933	71.7 (35.4)	70 [46–96]	29	3.1	3.2 [2.4–3.9]	3.1 (1.8)	40	4.3	4.3 [3.3–9.7]	5.7 (3.7)
		Overall	1449	111.5 (30.8)	112 [85–141]	43	3	2.4 [1.6–3.7]	2.8 (1.7)	75	5.2	4.9 [3.5–8.2]	5.9 (3.5)
	Putamen	SOX10+	533	44.4 (18)	49.5 [35.8–55.8]	17	3.2	2.0 [1.6–4.4]	3 (2.6)	34	6.4	6.4 [4.1–7.8]	6.2 (3.7)
		SOX10-	539	44.9 (18)	42.5 [34–55.5]	17	3.2	2.3 [1.3–3.4]	3.7 (5.4)	52	9.6	8.8 [8.0–13.2]	9.6 (4.5)
		Overall	1072	89.3 (26.1)	97.5 [80–99]	34	3.2	2.1 [1.7–4.6]	3.7 (3.9)	86	8	8.2 [7.0–10.4]	8.2 (3)
	SN	SOX10+	495	38.1 (16.7)	35 [26–42]	12	2.4	2.4 [0–3.4]	2.5 (3)	40	8.1	6.5 [3.8–10.3]	8.5 (6)
		SOX10-	569	43.8 (25.8)	37 [24–49]	29	5.1	4.8 [2.6–6.1]	4.6 (3.5)	49	8.6	9.1 [6.7–12.5]	9.7 (5.7)
		Overall	1064	81.8 (26.1)	81 [64–95]	41	3.9	3.7 [1.8–5.3]	4.1 (4.3)	89	8.4	8.1 [6.0–11.1]	9.4 (4.6)
MSA-SND	Cerebellum	SOX10+	626	56.9 (22.4)	58 [49.5–64.5]	32	5.1	3.8 [2.7–5.8]	5.2 (4.7)	77	12.3	13.0 [11.5–14.2]	11.9 (3.7)
		SOX10-	344	31.3 (18.6)	24 [21–36]	6	1.7	0 [0–5.4]	3.2 (6.2)	51	14.8	14.7 [8.7–18.2]	15.5 (9.5)
		Overall	970	88.2 (22)	82 [78.5–95.5]	38	3.9	2.5 [1.6–7.4]	7.5 (5)	128	13.2	12.7 [10.4–16.7]	13.4 (3.2)
	Putamen	SOX10+	630	57.3 (24.7)	55 [40–75]	51	8.1	7.3 [5.6–7.9]	8.5 (6.4)	96	15.2	13.7 [11.9–15.3]	14.8 (7.9)
		SOX10-	377	34.3 (11.1)	35 [30.5–40]	25	6.6	5.7 [3.0–6.4]	6 (4.9)	55	14.6	15.6 [8.5–20]	15.2 (8.5)
		Overall	1007	91.5 (27)	87 [81.5–107.5]	76	7.5	6.8 [4.9–7.1]	7.5 (5)	151	15	14.2 [11.5–17.8]	15.1 (6.6)
	SN	SOX10+	524	58.2 (18.5)	54 [46–74]	35	6.7	6.4 [4.5–7.5]	7 (4.3)	77	14.7	11.9 [11.1–15.5]	13.8 (4.7)
		SOX10-	286	31.8 (19.4)	24 [21–35]	16	5.6	4.2 [0–9.5]	5.8 (5.9)	41	14.3	10.5 [8.3–18.2]	12.5 (7.5)
		Overall	810	90 (17.2)	101 [76–102]	51	6.3	5.3 [4.9–5.9]	6.3 (3.1)	118	14.6	13.2 [10.4–16.7]	14.1 (4.8)
MSA-OPCA	Cerebellum	SOX10+	380	34.5 (16.5)	26 [23.5–47.5]	42	11.1	8.7 [7.3–19.1]	12.2 (8.7)	62	16.3	15.4 [13.8–18.4]	16.6 (4.1)
		SOX10-	541	49.2 (22.6)	44 [31–66]	47	8.7	7.9 [4.5–12.3]	8.5 (5.5)	55	10.2	10.9 [6.3–14.3]	10.8 (6)
		Overall	921	83.7 (21.1)	88 [70–90.5]	89	9.7	7.9 [6.8–10.5]	9.4 (4.5)	117	12.7	12.9 [10.8–14.5]	13.3 (4.3)
	Putamen	SOX10+	443	49.2 (38.9)	28 [21–60]	30	6.8	9.5 [3.6–11.4]	7.9 (4.8)	58	13.1	15.0 [10.5–15.9]	12 (5.9)
		SOX10-	425	47.2 (19)	41 [36–55]	41	9.7	8.3 [4.5–13.1]	9.2 (5.8)	46	10.8	11.1 [9.1–12.2]	9.9 (5)
		Overall	868	96.4 (38)	85 [83–113]	71	8.2	9.9 [3.6–12.4]	8.8 (5.5)	104	12	10.6 [9.5–14.1]	11.3 (3.4)
	SN	SOX10+	453	45.3 (21.6)	45 [34–55]	37	8.2	8.4 [6.1–13.0]	10 (6.7)	45	9.9	8.0 [7.3–13.6]	10.3 (5.3)
		SOX10-	466	46.6 (26.9)	38 [30.5–60]	30	6.5	4.7 [3.3–9.2]	6.3 (4.6)	61	13.1	14.1 [8.3–15.4]	13.7 (6.7)
		Overall	919	91.9 (24.5)	98.5 [72–109]	67	7.3	6.3 [4.2–11.3]	7.6 (4.2)	106	11.5	10.4 [8.0–13.8]	11.8 (5.1)

Values represent the total number of cells analysed and numbers/percentages of cells with CNV gains or losses, including medians [IQR] and means (±SD). *Abbreviations:* SN, substantia nigra; CNV, copy number variant; SND, striatonigral degeneration; OPCA, olivopontocerebellar atrophy; SD, standard deviation; IQR, interquartile range.

(a)

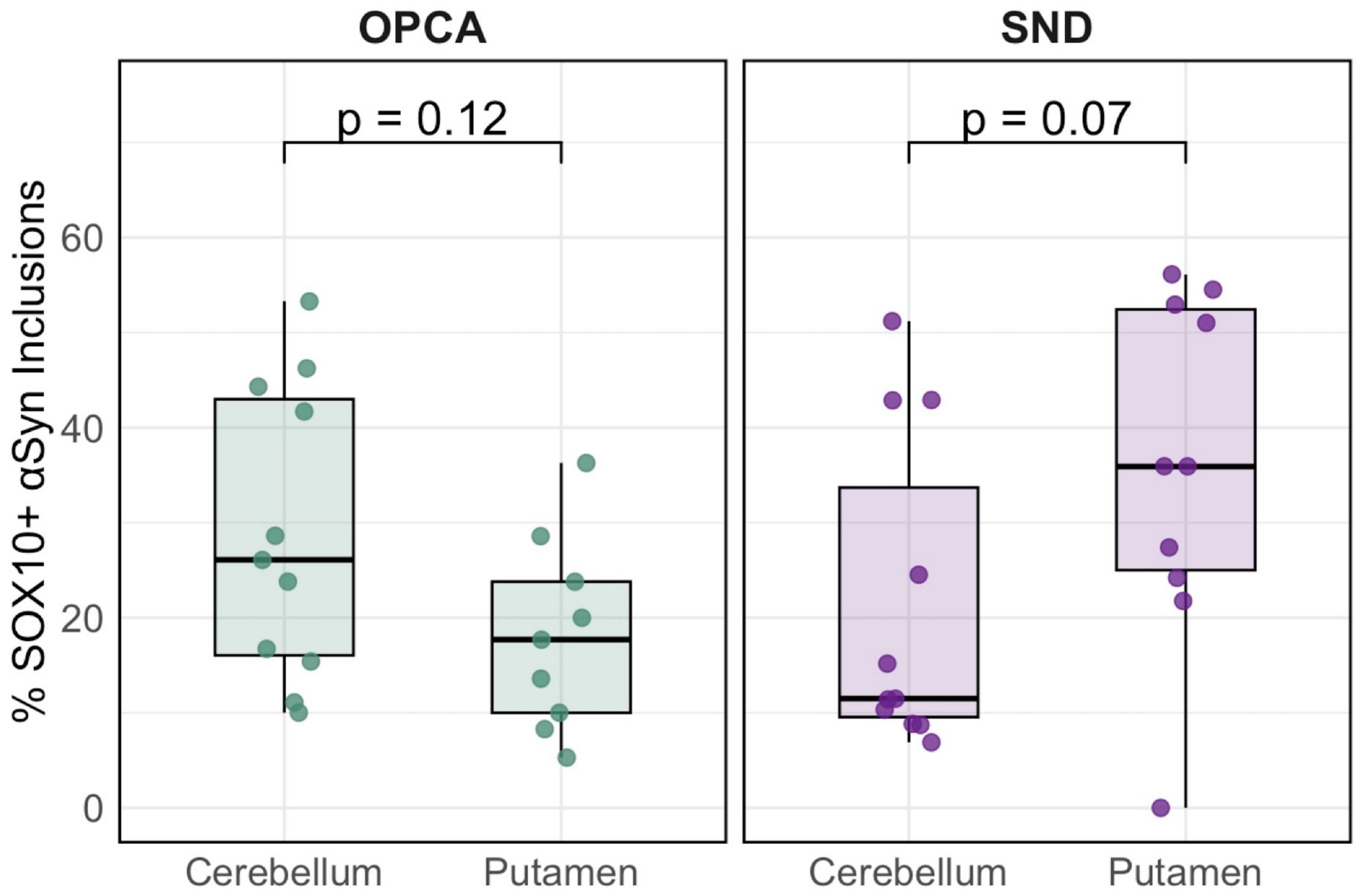

(b)

Subtype	Region	Median (%) SOX10⁺ αSyn⁺ [IQR]
OPCA	Cerebellum	26.1 [16.0–43.0]
	Putamen	17.7 [10.0–23.8]
	SN	25.6 [17.2–36.2]
SND	Cerebellum	11.5 [9.6–33.7]
	Putamen	35.9 [25.0–52.4]
	SN	15.0 [11.3–19.3]

Supplementary Fig 3. Regional distribution of $SOX10^{+}$ α-synuclein^{+} inclusions in MSA subtypes. (A) Comparison of $SOX10^{+}$ α-synuclein^{+} cells across preferentially affected versus less affected regions in SND and OPCA. P-values were calculated using the Mann–Whitney U test. (B) Corresponding summary table of inclusion data presented as median (%) [IQR] for each region and MSA subtype.

Supplementary Table 3. Distribution of *SNCA* copy number status according to α-synuclein inclusion status.

Region	MSA subtype	Donors (n)	αSyn status	Gain n (%)	Loss n (%)	CN2 n (%)	Total
**SOX10+ cells**							
**Cerebellum**	OPCA	11	αSyn+	26 (22.6)	18 (15.7)	71 (61.7)	115
			αSyn−	16 (6.0)	44 (16.6)	205 (77.4)	265
	SND	11	αSyn+	2 (1.5)	21 (15.3)	114 (83.2)	137
			αSyn−	30 (6.1)	56 (11.5)	403 (82.4)	489
**Putamen**	OPCA	9	αSyn+	7 (7.7)	14 (15.4)	70 (76.9)	91
			αSyn−	23 (6.5)	44 (12.5)	285 (81.0)	352
	SND	10	αSyn+	32 (16.1)	31 (15.6)	136 (68.3)	199
			αSyn−	16 (4.5)	41 (11.4)	302 (84.1)	359
**SN**	OPCA	10	αSyn+	16 (13.9)	19 (16.5)	80 (69.6)	115
			αSyn−	21 (6.2)	26 (7.7)	291 (86.1)	338
	SND	8	αSyn+	9 (10.3)	19 (21.8)	59 (67.8)	87
			αSyn−	21 (5.8)	41 (11.3)	301 (82.9)	363
Total			αSyn+	92 (12.4)	132 (17.7)	530 (71.2)	744
			αSyn−	127 (5.9)	252 (11.6)	1787 (82.5)	2166
**SOX10- cells**							
**Cerebellum**	OPCA	11	αSyn+	7 (23.3)	5 (16.7)	18 (60.0)	30
			αSyn−	40 (7.8)	50 (9.8)	421 (82.4)	511
	SND	11	αSyn+	0 (0.0)	5 (25.0)	15 (75.0)	20
			αSyn−	6 (1.9)	37 (11.7)	272 (86.3)	315
**Putamen**	OPCA	9	αSyn+	1 (6.3)	4 (25.0)	11 (68.8)	16
			αSyn−	40 (9.8)	42 (10.3)	327 (80.0)	409
	SND	10	αSyn+	2 (6.5)	5 (16.1)	24 (77.4)	31
			αSyn−	22 (7.0)	44 (13.9)	250 (79.1)	316
**SN**	OPCA	10	αSyn+	2 (11.1)	6 (33.3)	10 (55.6)	18
			αSyn−	28 (6.3)	55 (12.3)	365 (81.5)	448
	SND	8	αSyn+	0 (0.0)	3 (30.0)	7 (70.0)	10
			αSyn−	16 (6.4)	32 (12.9)	201 (80.7)	249
**Total**			αSyn+	12 (9.6)	28 (22.4)	85 (68.0)	125
			αSyn−	152 (6.8)	260 (11.6)	1836 (81.7)	2248

Distribution of *SNCA* copy number gain, loss, and copy number 2 (CN2) in α-synuclein-positive (αSyn+) and α-synuclein-negative (αSyn−) $SOX10^{+}$ and $SOX10^{-}$ nuclei across MSA brain regions and subtypes. Values are shown as n (%), with percentages calculated within each αSyn group.

Supplementary Table 4. Spearman correlations between *SNCA* CNVs, α-synuclein inclusions, and demographic variables in MSA and control brains.

Variable 1	Variable 2	Group	Rho	p-value
% $SOX10^+$ *SNCA* Gains (All)	% $SOX10^-$ *SNCA* Gains (All)	MSA	**0.5**	**< 0.0001***
		Control	0.22	0.17
% $SOX10^+$ *SNCA* Gains (MA)	% $SOX10^+$ *SNCA* Gains (LA)	MSA	**0.56**	**0.02***
% $SOX10^+$ *SNCA* Gains (CER)	% $SOX10^+$ *SNCA* Gains (PUT)	Control	0.35	0.29
% $SOX10^+$ *SNCA* Losses (All)	% $SOX10^-$ *SNCA* Losses (All)	MSA	-0.002	0.99
		Control	0.28	0.09
% $SOX10^+$ *SNCA* Losses (MA)	% $SOX10^+$ *SNCA* Losses (LA)	MSA	**0.7**	**0.002***
% $SOX10^+$ *SNCA* Losses (CER)	% $SOX10^+$ *SNCA* Losses (PUT)	Control	0.2	0.47
% $SOX10^+$ *SNCA* Gains (MA)	% $SOX10^+$ α-synuclein inclusions (MA)	MSA	**0.49**	**0.02***
% SOX10- *SNCA* Gains (MA)	% $SOX10^-$ α-synuclein inclusions (MA)	MSA	0.17	0.44
% $SOX10^+$ *SNCA* Losses (MA)	% $SOX10^+$ α-synuclein inclusions (MA)	MSA	-0.3	0.1
% $SOX10^-$ *SNCA* Losses (MA)	% $SOX10^-$ α-synuclein inclusions (MA)	MSA	0.26	0.25
Average % $SOX10^+$ *SNCA* Gains	Age of death (years)	MSA	-0.34	0.09
		Control	-0.1	0.72
	Age of onset (years)	MSA	**-0.45**	**0.03***
		Control	NA	NA
	PMI (hours)	MSA	0.27	0.19
		Control	0.31	0.27
	Disease duration (years)	MSA	0.10	0.63
		Control	NA	NA
Average % $SOX10^-$ *SNCA* Gains	Age of death (years)	MSA	-0.12	0.57
		Control	-0.15	0.59
	Age of onset (years)	MSA	-0.11	0.58
		Control	NA	NA
	PMI (hours)	MSA	0.10	0.58
		Control	0.29	0.29
	Disease duration (years)	MSA	0.17	0.45
		Control	NA	NA
Average % $SOX10^+$ *SNCA* Losses	Age of death (years)	MSA	-0.19	0.35
		Control	0.3	0.25
	Age of onset (years)	MSA	-0.06	0.79
		Control	NA	NA
	PMI (hours)	MSA	-0.09	0.68
		Control	-0.18	0.54
	Disease duration (years)	MSA	0.11	0.61
		Control	NA	NA
Average % $SOX10^-$ *SNCA* Losses	Age of death (years)	MSA	-0.02	0.92
		Control	0.21	0.45
	Age of onset (years)	MSA	-0.2	0.34
		Control	NA	NA
	PMI (hours)	MSA	-0.04	0.84
		Control	-0.07	0.8
	Disease duration (years)	MSA	0.26	0.23
		Control	NA	NA

Spearman correlation coefficients (Rho) and p-values are shown for relationships between *SNCA* gains and losses in $SOX10^+$ and $SOX10^-$ cells, α-synuclein inclusions and demographic variables (age at death, age of onset, post-mortem interval [PMI]). Analyses are shown for all regions, specific brain regions (cerebellum [CER], putamen [PUT], most affected [MA], and lesser affected [LA] regions), and averages across regions. Significant correlations ($p < 0.05$) are indicated with an asterisk (*) and are in bold. NA= not applicable.

Supplementary Table 5. Summary of MSA and Control demographics for γh2ax staining

Group	n	Age at death (years)		PMI (hours)		Age at disease onset (years)		Disease duration (years)		Sex (M/F)
		Mean ± SD	Median [IQR]	Mean ± SD	Median [IQR]	Mean ± SD	Median [IQR]	Mean ± SD	Median [IQR]	
Control	4	64 ± 8.1	64.5 [57.5–71]	74.7 ± 26.5	79 [66.7–87]	n.a.	n.a.	n.a.	n.a.	2 (50%) / 2 (50%)
MSA (SND + OPCA)	10	62.7 ± 5.8	64 [58.8–67]	64.8 ±20.9	63.2 [53.2-72.8]	56 ± 4.3	57 [55-57]	7.2 ± 2.4	7 [6-7]	5 (50%) / 5 (50%)
p-value		Mann Whitney U-test p = 0.57		Mann Whitney U-test p = 0.44		n.a.		n.a.		Fisher's exact p = 1

Demographic variables are shown for control cases and MSA cases, with SND and OPCA combined. Data are presented as mean ± standard deviation (SD) and median with interquartile range (IQR), as indicated. PMI, post-mortem interval.

Supplementary Table 6. Summary of donors used and regions analysed per γH2AX staining experiment

Staining Experiment	Donor	Regions
**SOX10 + γH2AX**	Control10	Cerebellum, Putamen
	Control11	Cerebellum, Putamen
	Control12	Substantia Nigra
	Control14	Putamen
	OPCA02	Putamen
	OPCA04	Cerebellum, Substantia Nigra
	OPCA05	Putamen
	OPCA10	Putamen
	OPCA12	Cerebellum, Putamen
	SND03	Cerebellum, Putamen, Substantia Nigra
	SND05	Cerebellum, Putamen
	SND11	Cerebellum, Putamen
	SND12	Cerebellum, Putamen, Substantia Nigra
	SND13	Cerebellum, Putamen
**αSyn + γH2AX**	Control10	Putamen, Substantia Nigra
	Control12	Cerebellum, Putamen
	Control14	Cerebellum, Substantia Nigra
	OPCA02	Cerebellum
	OPCA04	Putamen
	OPCA05	Cerebellum
	OPCA10	Cerebellum, Substantia Nigra
	SND03	Cerebellum
	SND11	Substantia Nigra

The table lists control, OPCA, and SND donors analysed by combined SOX10 or α-synuclein (αSyn) immunostaining with γH2AX, together with the brain regions examined for each donor.
